# New Crosslinked Single-Ion Silica-PEO Hybrid Electrolytes

**DOI:** 10.3390/polym14235328

**Published:** 2022-12-06

**Authors:** Sébastien Issa, Roselyne Jeanne-Brou, Sumit Mehan, Didier Devaux, Fabrice Cousin, Didier Gigmes, Renaud Bouchet, Trang N. T. Phan

**Affiliations:** 1 Institut de Chimie Radicalaire-UMR 7273, CNRS, Aix Marseille University, 13397 Marseille, France; 2 LEPMI, Grenoble INP, CNRS, Univ. Savoie Mont Blanc, Univ. Grenoble Alpes, 38000 Grenoble, France; 3 Laboratoire Léon Brillouin, Université Paris-Saclay, CEA-CNRS UMR 12, 91191 Gif-sur-Yvette, France

**Keywords:** crosslinked polymers, single-ion hybrid electrolytes, lithium metal batteries, sol–gel polycondensation, electrochemical impedance spectroscopy

## Abstract

New single-ion hybrid electrolytes have been synthetized via an original and simple synthetic approach combining Michael addition, epoxidation, and sol–gel polycondensation. We designed an organic PEO network as a matrix for the lithium transport, mechanically reinforced thanks to crosslinking inorganic (SiO_1.5_) sites, while highly delocalized anions based on lithium vinyl sulfonyl(trifluoromethane sulfonyl)imide (VSTFSILi) were grafted onto the inorganic sites to produce single-ion hybrid electrolytes (HySI). The influence of the electrolyte composition in terms of the inorganic/organic ratio and the grafted VSTFSILi content on the local structural organization, the thermal, mechanical, and ionic transport properties (ionic conductivity, transference number) are studied by a variety of techniques including SAXS, DSC, rheometry, and electrochemical impedance spectroscopy. SAXS measurements at 25 °C and 60 °C reveal that HySI electrolyte films display locally a spatial phase separation with domains composed of PEO rich phase and silica/VSTFSILi clusters. The size of these clusters increases with the silica and VSTFSILi content. A maximum ionic conductivity of 2.1 × 10^−5^ S·cm^−1^ at 80 °C has been obtained with HySI having an EO/Li ratio of 20. The Li^+^ ion transfer number of HySI electrolytes is high, as expected for a single-ion electrolyte, and comprises between 0.80 and 0.92.

## 1. Introduction

To accelerate the growth of renewable energy production and electric mobility, a tremendous challenge is to develop safe, efficient and cheap battery systems [1,2,3]. Among the foreseen battery technologies, lithium (Li) metal-based batteries are very appealing thanks to the high theoretical specific capacity of Li (3.86 A·h·g^−1^), ten times higher than that of conventional graphite-based negative electrodes widely used in Li-ion batteries. In addition, lithium metal has a very low standard potential [4] (−3.04 V vs. standard hydrogen electrode) that promises high specific energy densities for lithium metal battery technology. However, its application in rechargeable batteries in the presence of organic-based liquid electrolytes (carbonates and ethers) is hindered by the poor coulombic efficiency and safety issues caused by uneven Li plating/stripping [5,6,7,8]. To solve this problem, inorganic and polymer solid-state electrolytes are key materials [9]. Inorganic electrolytes typically show high ionic conductivity (10^−4^–10^−2^ S·cm^−1^) at room temperature and mechanical strength, while polymer electrolytes provide flexibility, processability and form intimate contact with the electrodes. However, many issues still need to be addressed as inorganic electrolytes have low adhesion to electrode-active materials, are too brittle to compensate for the electrode volume changes during cycling and are difficult to manufacture, which hinders their practical use [10]. For polymer electrolytes, their low ionic conductivity at room temperature renders them incapable of achieving the required charging/discharging battery rate [10]. Therefore, the development of a solid electrolyte comprising all the needed properties, such as high ionic conductivity while being a single-ion conductor, a wide electrochemical stability window, excellent thermal stability and mechanical strength, a low cost combined with a synthesis easiness would be a major advancement to promote all solid Li metal batteries [11].

An efficient approach combining the advantages of inorganic and polymer electrolytes while overcoming their disadvantages lies in their hybridization. According to the literature, the main strategy to prepare hybrid solid electrolytes is to disperse inorganic fillers of high surface area into a polymer matrix [12]. Amongst polymer electrolytes, poly(ethylene oxide) (PEO)-based electrolytes are extensively studied due to their excellent salt solvation and low glass transition temperature. However, PEO is semi-crystalline by nature with a melting temperature of around 60 °C leading to low ionic conductivity at room temperature. In addition, the Li^+^ transference number (*t*^+^) of PEO-based electrolytes is also low because of the strong coordination of the Li-ion by the ether oxygen sites. A high *t*^+^ is desired as a low value induces a concentration gradient of mobile species limiting the output power of the battery and favoring dendrite nucleation [13]. Concerning inorganic fillers, they can be divided into two main categories based on their functionalities: inert fillers and ion-conducting fillers. Inert fillers, including ceramic nanoparticles such as Al_2_O_3_, TiO_2_ and SiO_2,_ were widely studied by Croce et al. [14,15,16] at the end of the 1990s. Even though these inorganic oxides do not participate in ion conduction directly, it was reported that the addition of fillers to PEO-based electrolytes increased ionic conductivity, especially below the melting temperature, as it decreased the degree of crystallinity, and widened the electrochemical stability window [17]. Ceramic-based nanoparticles also influence the mechanical properties of the electrolytes. Typically, Young’s modulus increased from 500 to 2300 Pa with the addition of 10% SiO_2_ in a poly(vinyl chloride)-PEO electrolyte [18].

Although many hybrid solid electrolytes demonstrated better ionic conductivity than that of the polymer electrolyte matrix itself, [19] one challenge lies in the quality of the filler dispersion within the polymer matrix, as strong particle agglomeration can occur. The best strategy to obtain a good dispersion is to create a covalent link between the fillers and the polymer. Indeed, Vélez et al. [20] described a new route to prepare covalent organic–inorganic hybrid solid electrolytes by using a combination of the sol–gel method and organic polymerization. The maximum conductivity found for these hybrids is 2.6 × 10^−5^ S·cm^−1^ at room temperature with an electrochemical stability window up to 5.5 V vs. Li^+^/Li, but with a *t*^+^ of 0.37.

To address this challenge, we developed a hybrid solid electrolyte that combines enhanced thermal and mechanical properties with high single-ion conductivity. This electrolyte is composed of a PEO matrix ensuring ionic motion, inorganic (SiO_1.5_) crosslinking sites reinforcing the mechanical properties and highly delocalized grafted anions for high transport numbers. It results in a homogenous crosslinked organic–inorganic hybrid material. The interest of these hybrid electrolytes resides not only in their physicochemical properties but also in the facile synthesis route proposed. The synthesis was based on a simple three-step reaction in one pot without the use of hazardous solvents. The influence of the electrolyte composition, in terms of the inorganic/organic ratio and grafted anion content on the local structural organization, and the thermal, mechanical and electrochemical properties (ionic conductivity, *t*^+^), are studied to establish the full extent of the structure–property relationships. 

## 2. Experimental Section

**Materials.** Poly(ethylene glycol) (PEG, M_n_ = 4000 g·mol^−1^), epichlorohydrin (99%), sodium hydride (60% dispersion in mineral oil), (3-aminopropyl)triethoxysilane (APTES, 98%), Amberlite^®^ IRC120 H (hydrogen form), lithium hydroxide (>98%), acetonitrile (99.5%) and anhydrous ethanol (99.5%) were purchased from Sigma-Aldrich (Saint-Quentin Fallavier, France) and used as received. 2-chloroethanesulfonyl chloride (96%) and trifluoromethanesulfonamide (>98%) were purchased from TCI (Zwijndrecht, Belgium). The triethylammonium vinyl sulfonyl(trifluoromethane sulfonyl)imide (VSTFSI-triethylammonium) was prepared as described in our previous work [21]. All solvents and other reagents were synthesis-grade products and used without further purification.

**Nuclear magnetic resonance (NMR).** ^1^H, ^19^F and ^7^Li NMR spectra were recorded on a Bruker AVANCE 400 MHz machine using deuterated solvents such as CDCl_3_ or DMSO-*d*_6_.

**Synthesis of α,ω-diepoxy-PEO (diepoxy–PEO).** The diepoxy–PEO was prepared according to a procedure in the literature with some adaptations [22]. Briefly, α,ω-dihydroxyl poly(ethylene glycol) of 4000 g·mol^−1^ (25.0 g, 6.23 mmol) was dissolved in 300 mL of toluene and dried with azeotropic distillation using a Dean–Stark apparatus. The hydroxyl end-groups of the PEG were then converted into sodium alkoxide by its reaction with sodium hydride (10 eq, 2.5 g, 62.30 mmol) at 40 °C for 2 h. After this time, epichlorohydrin (12 eq, 6.0 mL, 74.76 mmol) was slowly added to the solution, and the mixture was stirred at 40 °C overnight. After the reaction, the mixture was filtered and the polymer was precipitated in diethyl ether, filtered, washed with diethyl ether and dried under vacuum at room temperature. The coupling yield was quantitative. ^1^H NMR (400 MHz, CDCl_3_) δ (ppm): 3.90–3.35 (m, 363H, -CH_2_-CH_2_-O-), 3.15 (m, 2H, CH of epoxide ring), 2.78 and 2.60 (m, 4H, CH_2_ of epoxide ring).

**Synthesis of lithium vinyl-STFSI (VSTFSILi).** Amberlite ^®^IRC 120-H form ion-exchange resin (150 g) was successively washed with deionized water (2 × 500 mL), 1M LiOH (3 × 500 mL) and deionized water (6 × 500 mL) until a neutral pH was obtained. The resin beads were then loaded into a column of 3.5 cm outside diameter. Elution across the column of 10 g of triethylammonium vinyl-STFSI dissolved in 150 mL of water/ethanol (95:5, *v*/*v*) mixture, producing an aqueous solution of vinyl-STFSILi. The outflow was set at approximately one drop per second to enhance the cation exchange. This operation is repeated two more times with new Amberlite IR120 Li (50 g). The resulting solution was washed with dichloromethane (3 × 100 mL) to remove any traces of triethylammonium vinyl-STFSI. Water and ethanol were removed using a rotary evaporator at 50 °C. The lithium vinyl-STFSI was dried under vacuum at 60 °C for 24 h and stored under argon. Yield: 95%. ^1^H NMR (300 MHz, DMSO-d_6_, *δ* ppm): 6.72 (dd, J = 16.1, 10.4 Hz, 1H), 5.90 (d, J = 16.6 Hz, 1H), 5.72 (d, J = 9.9 Hz, 1H).^19^F NMR (376 MHz, DMSO-d_6_, δ ppm): −77.64.

**Figure 1:**
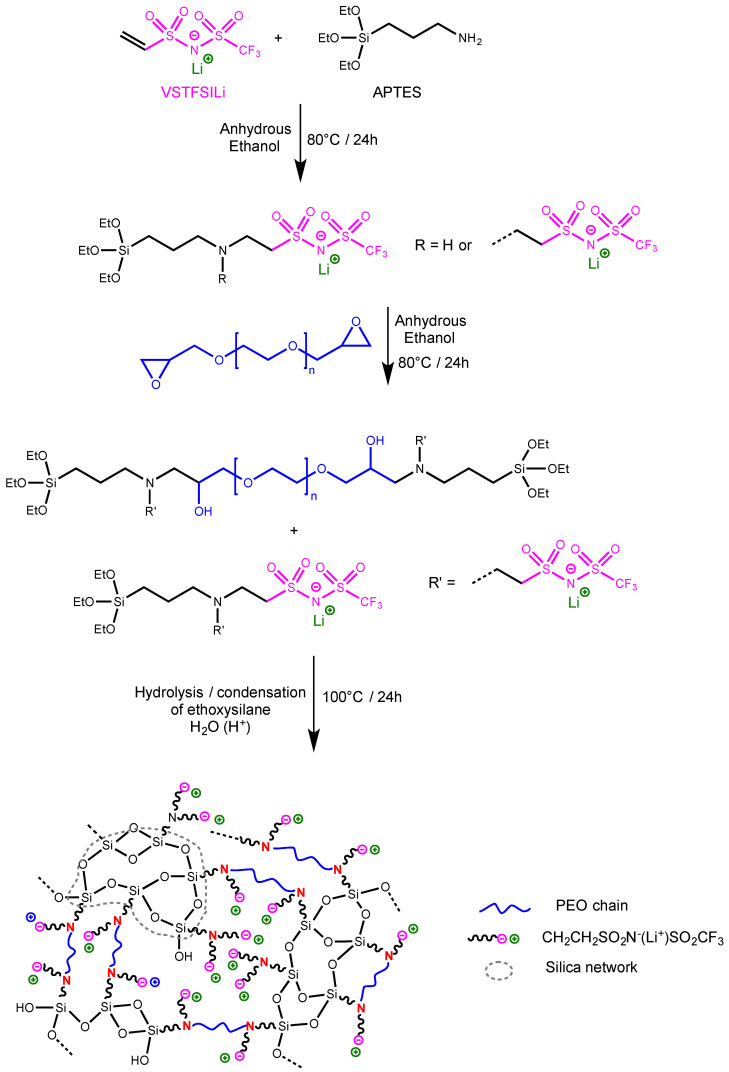
Synthesis route of hybrid single-ion electrolytes.

**Synthesis of Hybrid Single-Ion conducting electrolyte (HySI).** The electrolytes were prepared using a sol–gel process involving diepoxy–PEO, lithium vinyl-STFSI (VSTFSILi) and APTES (Figure 1). Typically, VSTFSILi (0.16300 g, 0.665 mmol, 1.4 eq) and anhydrous ethanol (1 mL) are introduced in a round bottom flask. The resulting solution was then heated at 80 °C. (3-aminopropyl)triethoxysilane (0.0946 mL, 0.479 mmol, 2.0 eq) was added dropwise to the solution and the mixture was stirred at 80 °C for 24 h. Then, diepoxy–PEO (0.58511 g, 0.146 mmol, 0.6 eq) dissolved in 1 mL of anhydrous ethanol was added to the reaction mixture. After complete addition, the reaction was continued for 24 h. After this time, anhydrous ethanol (10 mL) and 25.9 µL of acidic water at pH = 1.5 were subsequently added to the reaction mixture and the reaction continued for 1 h. After this time, the reaction mixture was poured into a PTFE dish and crosslinked at room temperature for 1 h, then at 100 °C for 24 h. The resulting HySI are homogeneous, transparent and crack-free films with good mechanical strength and a good degree of elasticity. Their thickness is comprised between 35 µm and 90 µm. Table 1 summarizes all experimental data used for the synthesis of various hybrid single-ion electrolytes.

For each electrolyte, the weight fraction of silica species, denoted by SiO_1.5_, was calculated by assuming each silicon atom was surrounded by three oxygen atoms and one alkyl group in the hybrid:

(1) $$w_{{\mathrm{SiO}}_{1.5}}=\frac{M_{{\mathrm{SiO}}_{1.5}}\times n_{\mathrm{APTES}}}{(M_{{\mathrm{SiO}}_{1.5}}\times n_{\mathrm{APTES}})+(M_{\mathrm{aminopropyl}}\times n_{\mathrm{APTES}})+m_{\mathrm{PEO}}+m_{\mathrm{VSTFSILi}}}$$

where $M_{{\mathrm{SiO}}_{1.5}}$ is the SiO_1.5_ molecular mass (52 g·mol^−1^), *n*_APTES_ is the molar number of APTES, *M*_propylamine_ is the molecular mass of the aminopropyl of the APTES (58 g·mol^−1^), *m*_PEO_ is the PEO mass, and *m*_VSTFSILi_ is the mass of the lithium vinyl-STFSI.

Different electrolytes were prepared by varying the lithium content (EO/Li ratio) and, thus, the inorganic/PEO ratios. For clarity, the HySI electrolytes are denoted by HySI_*w*_VSTFSILi_, with *w*_VSTFSILi_ as the VSTFSILi weight fraction (in percent) throughout the text.

Table 2 summarizes the composition of the produced HySI electrolytes. For each sample, the sum of *w*_VSTFSILi_ and the PEO (*w*_PEO_) and SiO_1.5_ (*w*_SiO_1.5__) weight fractions is not equal to 100% due to the presence of APTES aminopropyl residual fragments. In addition, two additional series of hybrid electrolytes were synthesized to focus on the understanding of the relationships between chemistry structure and electrolyte properties. The first one has the same silica composition as HySI_20 but is lithium-free (HySI_0, Table 1, entry 1) and the second one has a similar lithium concentration as HySI_20 but contains double the silica. These electrolytes were synthesized by adding to the reaction mixture either triethoxymethyl silane (TEMS) (Table 1, entry 5) or tetraethoxysilane (TEOS) (Table 1, entry 6), and are denoted by HySI_20_TEOS and HySI_20_TEMS, respectively. The compositions of these last three materials are also added to Table 2.

**Differential Scanning Calorimetry (DSC).** The analysis of the thermodynamic behavior of the HySI electrolytes was conducted on a Mettler Toledo GC 20 TA DSC using a heat/cool/heat cycle from −100 °C to 125 °C and at a heating and cooling rate of 10 °C·min^−1^. Since the sample thermal history can alter the results, the first cycle was used to place all the electrolytes in the same state (similar crystallization conditions); then, the second and subsequent cycles were reproducible. The second heating cycle was used to analyze the thermogram and determine first the glass transition temperature (*T*_g_) of the HySI electrolytes with the tangent line methods. Then, from the PEO endothermic peak, the PEO melting temperature (*T*_m_) was determined as being the intersection point of the signal baseline and the inflectional tangent at the beginning of the melting peak. The PEO degree of crystallinity (*χ*_c_) was calculated from the melting enthalpy (Δ*H*_m_, determined by melting peak integration) and weight fraction of PEO (*w*_PEO_) according to:

(2) $$\chi_{c}=\frac{\Delta H_{m}}{w_{\mathrm{PEO}}\;.\;\Delta H_{m}^{0}}\cdot 100$$

where $\Delta H_{m}^{0}$ is the melting enthalpy of a 100% crystalline PEO taken as 195 J.g^−1^ [23].

**Rheometric experiments.** The viscoelastic properties of the HySI electrolytes were studied using an Anton Paar MCR 302 (Modular Compact Rheometer) equipped with 8 mm diameter disposable aluminum parallel disks. Before the measurements, the samples were stored in a desiccator containing calcium chloride to remove any trace of residual water. The samples were maintained at 75 °C—well above *T*_m_—and were pressed to a gap width of around 0.8 mm under a constant flow of dry air. The storage modulus (G′) and loss modulus (G″) were measured as a function of frequency, from 0.1 to 100 rad·s^−1^, by dynamically shearing the electrolyte at a fixed strain of 1%.

**Small- and Wide-Angle X-ray Scattering (SAXS/WAXS).** Measurements were carried out on a Xeuss 2.0 HR SAXS/WAXS instrument from Xenocs. The instrument uses a microfocused Cu Kα source with a wavelength of 1.54 Å and a PILATUS3 detector (Dectris). The experiments were performed at two sample-to-detector distances, 2490 mm with a collimated beam size of 0.5 × 0.5 mm and 333 mm with a collimated beam size of 0.8 × 0.8 mm, respectively, allowing them to achieve a broad scattering vector (*q*) range of 0.004–1.8 Å^−1^, with a good overlap between the two configurations. Electrolyte films were molded into a polypropylene ring and sealed between two Kapton sheets. Samples were placed onto a homemade sample holder thermalized with a circulating water flow coupled with a Huber bath, allowing control of the temperature of the samples up to 65 °C. Samples’ transmissions were measured precisely. Scattering from the empty beam, Kapton sheets and darkfield was measured independently and subtracted from the samples’ scattering according to standard procedures to obtain scattering in absolute units (cm^−1^). All samples were measured over the following temperature cycle: first at 25 °C, then heated up at 60 °C and cooled back to 25 °C. A waiting time of 60 min was used between each step of the cycle to ensure the proper thermalization of the samples. 

**Li symmetric cell assembly.** The electrolytes were placed in an Argon-filled glove box (Campus, Jacomex, Dagneux, France) with ultralow water and O_2_ content (<1 ppm) for at least a week prior to any further experiments. There, an electrolyte disk (14 mm diameter) was placed onto a Li metal disk (10 mm diameter), and the two were co-laminated in temperature (80 °C) and pressure (3 bars) in a homemade calendering apparatus to ensure intimate interfacial contact. Another Li disk was subsequently placed onto the remaining uncovered electrolyte face, and the cell was calendered again. The resulting Li symmetric cell was then placed in a conventional CR2032 coin cell and sealed using a crimper (manual crimper, Hohsen, Tokyo, Japan).

**Ionic transport properties.** After assembly, the coin cells were taken out of the glove box and placed in a climatic chamber (Clima Temperatur Systeme) and connected to a multipotentiostat with impedance capabilities (VMP300, BioLogic, Seyssinet-Pariset, France). There, the cells were kept at 80 °C for a day. Then, electrochemical impedance spectroscopy (EIS) was performed at different temperatures from 80 °C to 100 °C by steps of 10 °C followed by a cooling scan down to 30 °C and a second heating scan from 35 to 95 °C, also by 10 °C steps. At each temperature, and after temperature equilibration, EIS was performed from 7 MHz to 0.3 Hz using an excitation voltage between 20 and 80 mV. The treatment of the EIS spectra is based on a subtractive methodology [24] detailed in the Section 3. This permits the extraction of the different contributions from the EIS spectra. Therefore, a resistance (*R*_i_) of a contribution i at a temperature (*T*) is linked to the ionic conductivity (*σ*_i_) according to:

(3) $$\sigma_{i}\left(T\right)=\frac{l}{S\cdot R_{i}\left(T\right)}$$

where *l* is the average electrolyte thickness measured after the experiments and *S* is the active surface area equivalent to that of the Li electrodes.

In this study, only the cooling and second heating scans are presented as the two data sets that are in agreement with each other. In addition, results are provided with a standard deviation calculated based on the measurements of at least three cell replicates per electrolyte composition. 

Moreover, as for PEO-based electrolytes, the VTF equation [25] is used to fit the *σ*-*T* relationship for *T* > *T*_m_ according to:

(4) $$\sigma \left(T\right)=\frac{\sigma_{0}}{\sqrt{T}}\cdot e^{\frac{-E_{a}}{R\cdot \left(T-T_{0}\right)}}$$

where *σ*_0_ is a pre-exponential factor proportional to the fraction of free ions, *E*_a_ is pseudo-activation energy, *R* is the ideal gas constant (8.314 J·K^−1^·mol^−1^) and *T*_0_ is the ideal glass transition temperature generally taken as *T*_g_—50.

Moreover, the dielectric constant (*ε*_i_) of a contribution i is calculated based on the corresponding capacity (*C*_i_) and the cell dimensions such as:

(5) $$\varepsilon_{i}=\frac{C_{i}\cdot l}{\varepsilon_{0\;}\cdot S}\;$$

where *ε*_0_ is the vacuum permittivity (8.854 10^−12^ F·m^−1^). 

*C*_i_ is calculated thanks to the equivalent circuit parameters used to fit the impedance of the contribution i, such as the resistance *R*_i_ and those from the Constant Phase Element (CPE), namely the pseudo-capacity (*Q*_i_) and the phase (*n*_i_) such as:

(6) $$C_{i}=R_{i}^{\frac{1}{n_{i}}-1}\;.\;Q_{i}^{\frac{1}{n_{i}}}\;$$

The Li symmetric coin cells were also used to determine the cationic transference number (*t*^+^) at 80 °C. For *t*^+^, the Bruce and Vincent methodology [26,27] was employed. The protocol consists in applying a small potentiostatic step, Δ*V* = 20 mV, to the cell and recording the current (*I*) until a steady-state current (*I*_ss_) is obtained, combined. In addition, an EIS spectrum is recorded before the polarization to determine the $R_{\mathrm{el}}$ of the electrolyte resistance and at the end of the polarization to determine the $R_{\mathrm{int}}$ of the final Li/electrolyte interface resistance.

A typical example of a polarization step performed at 80 °C on a Li symmetric cell comprising the HySI_33 electrolyte is provided in Figure S1. The transport number is calculated thanks to the equation proposed by Watanabe et al. [28]:

(7) $$t^{+}=\frac{R_{\mathrm{el}}}{\frac{V}{I_{\mathrm{SS}}}\;–\;R_{\mathrm{int}}}$$

## 3. Results and Discussion

The procedure used for the preparation of the crosslinked single-ion silica-PEO hybrid electrolyte (HySI) is a one-pot synthesis, depicted in Figure 1, based on a three-step reaction involving diepoxy–PEO, lithium vinyl-STFSI (VSTFSILi) and 3-Aminopropyltriethoxysilane (APTES) and using eco-friendly ethanol as solvent. In the first step (Figure 1), the Michael reaction between VSTFSILi and the amine function of APTES leads to APTES-lithium ethyl-STFSI intermediate product. In this kind of Michael addition reaction, it is well known that each primary amine of APTES can react with two VSTFSILi. In the present study, the molar ratio $r_{1}=\frac{2n_{APTES}}{n_{VSTFSILi}}$ (Table 1) between reactive functions of APTES and VSTFSILi varied from 1.22 to 1.66. Thus, at the end of the first step, we have statistically a mixture of APTES functionalized either with one or two STFSILi functions (Figure 1). The ^1^H NMR spectrum of the crude product (Figure S2) exhibits the presence of signals of the expected Michael adduct at 3.08 ppm labelled (g) and 2.83 ppm labelled (f) corresponding to the –CH_2_CH_2_SO_2_N^(−)^SO_2_CF_3_ anion. However, the signal of a secondary amine is difficult to observe because the NH bonds are polar and their protons are mobile. The presence of STFSI functionality was furthermore confirmed by ^19^F and ^7^Li NMR analysis with the fluorine and lithium signal at −77.57 ppm (Figure S3a) and at −1.05 ppm (Figure S3b), respectively. In the second step (Figure 1), the secondary amine groups of APTES-functionalized STFSILi react with epoxy functions of diepoxy–PEO at 80 °C for 24 h until the total disappearance of epoxy functions as verified by ^1^H NMR analysis (not shown here). In the last step, the hydrolysis of the alkoxysilane molecules was initiated by catalytic amounts of acidic water. The heat treatment allows the formation of -Si-O-Si- linkages and produces the silicate framework [20]. After a heat treatment at 100 °C for 24 h, flexible and self-supported crosslinked electrolyte membranes with thicknesses between 35 and 90 μm were obtained. Each HySI was conceived by fixing the targeted molar ratio of EO/Li and then calculating the number of APTES so that the molar ratio $r_{2}=\frac{2n_{APTES}}{\left(n_{VSTFSILi}+2n_{PEO}\right)}$ was equal to 1 (Table 1, entries 2–8). Thus, the concentration of SiO_1.5_ in hybrid electrolytes is indirectly conditioned by the EO/Li ratios. Various HySI were synthesized by varying EO/Li ratio and, thus, the SiO_1.5_ concentration.

**Figure 2:**
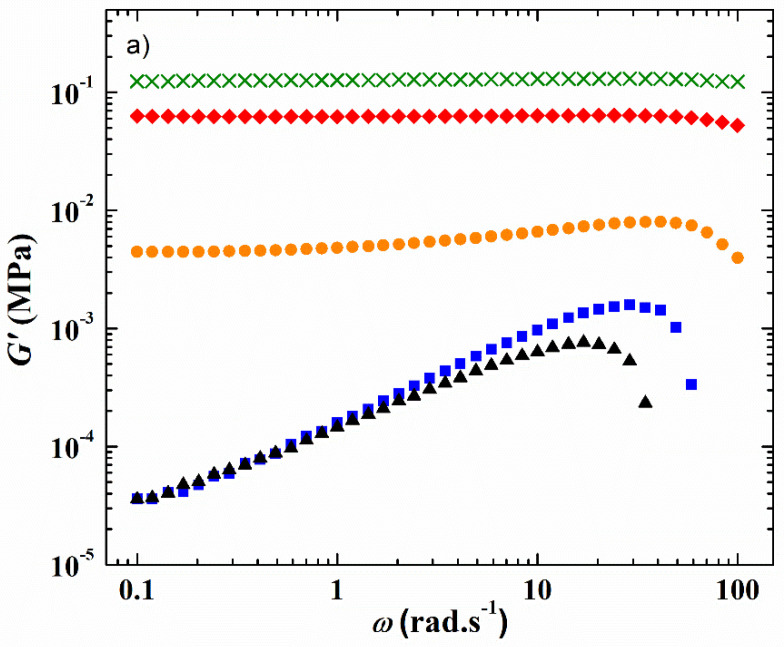

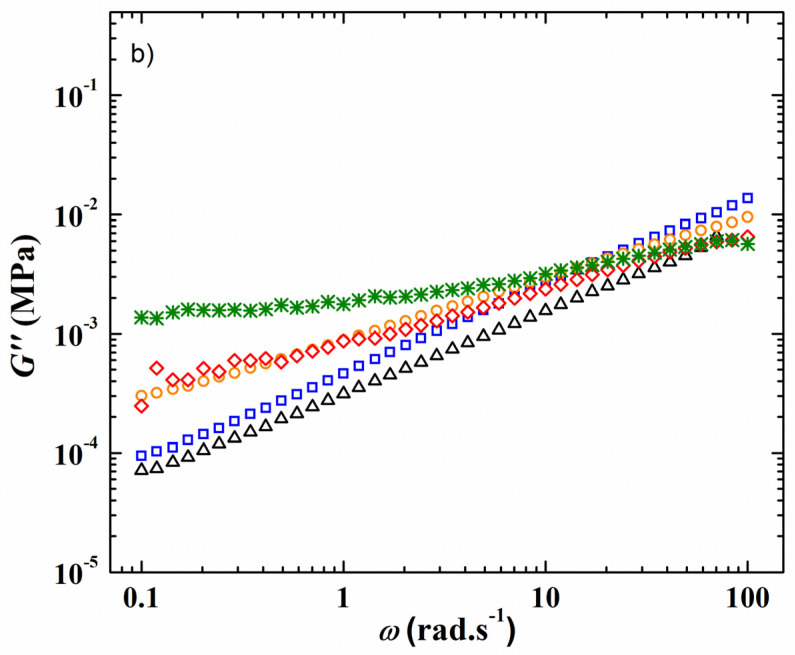
Frequency response at 75 °C and at a strain value fixed at 1% of (**a**) the storage modulus G′ and (**b**) the loss modulus G″ of the HySI electrolytes. The symbols correspond to *w*_VSTFSILi_ value of (green cross) 15, (red diamond) 17, (orange circle) 20, (black triangle) 25, and (blue square) 33.

**Figure 3:**
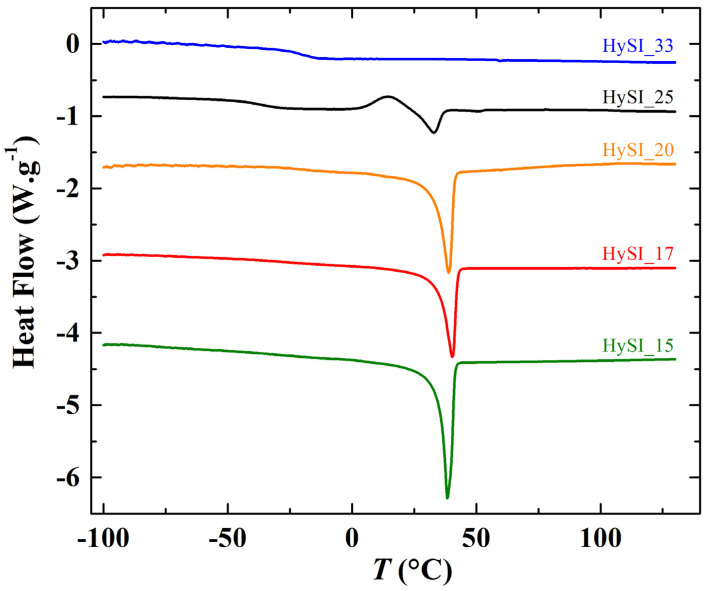
Thermograms from −100 °C to 125 °C at 10 °C·min^−1^ of the HySI electrolytes.

The viscoelastic properties at a constant strain of 1% of the HySI electrolytes corresponding to the variation of the storage G′ and loss G″ modulus at 75 °C as a function of the angular frequency (*ω*) are displayed in Figure 2a,b, respectively. At such a temperature, all the electrolytes are in a melted state (see Figure 3). For the HySI electrolytes with *w*_VSTFSILi_ ≤ 20, G′ is higher than G″ all over the studied angular frequency range, indicating solid-like viscoelastic behavior. In addition, at a given salt content, G′ is almost independent of *ω* due to the silica crosslinking sites that mitigate the stress relaxations in the hybrid materials. Moreover, G′ decreases with an increase of salt concentration, and thus silica content, typically from 0.12 MPa down to 5 kPa for *w*_VSTFSILi_ of 15 and 20, respectively. The loss modulus G″ increases slightly with *ω* with values in the range of 1 kPa. Conversely, for the highest concentrated electrolytes, HySI_25 and HySI_33, G′ is lower than G″, and both parameters increase with *ω* indicating liquid-like behavior. Such a difference in the viscoelastic behavior may be due to a smaller extent of crosslinking within these materials. Indeed, based on the SAXS/WAXS analysis, the two highest concentrated electrolytes are the more heterogeneous materials with large TFSI clusters surrounding the silica crosslinking sites. Therefore, the TFSI anion may prevent an overall high degree of crosslinking leading to liquid-like properties. 

In addition, as for the material thermal properties, the presence of Li salt has an important influence on the HySI viscoelastic properties due to its plasticizing effect. Similar to the literature data on PEO and PEO salt-in-polymer blends, [29,30] the comparison of the viscoelastic properties of HySI_0 and HySI_20, displayed in Figure S4, shows that G″ lies in the same range while G′ decreases by almost one order of magnitude when salt is present in the hybrid materials.

Figure 3 shows the DSC thermograms of different HySI electrolytes during the second heating scan. Typically, the thermograms present two characteristic transitions, one step at temperatures below 0 °C ascribed to the glass transition temperature (*T*_g_) and one endothermic peak at temperatures around 35 °C related to the PEO melting temperature. No melting peak was observed for the highest concentrated electrolyte (HySI_33), and for HySI_25, a small crystallization peak at about 14.5 °C was present only during the heating run, suggesting that its crystallization process was not complete during the cooling run. In addition, Figure S5 displays the thermograms of the HySI_0, HySI_20_TEMS and HySI_20_TEOS samples having a similar temperature profile than the other electrolytes.

**Figure 4:**
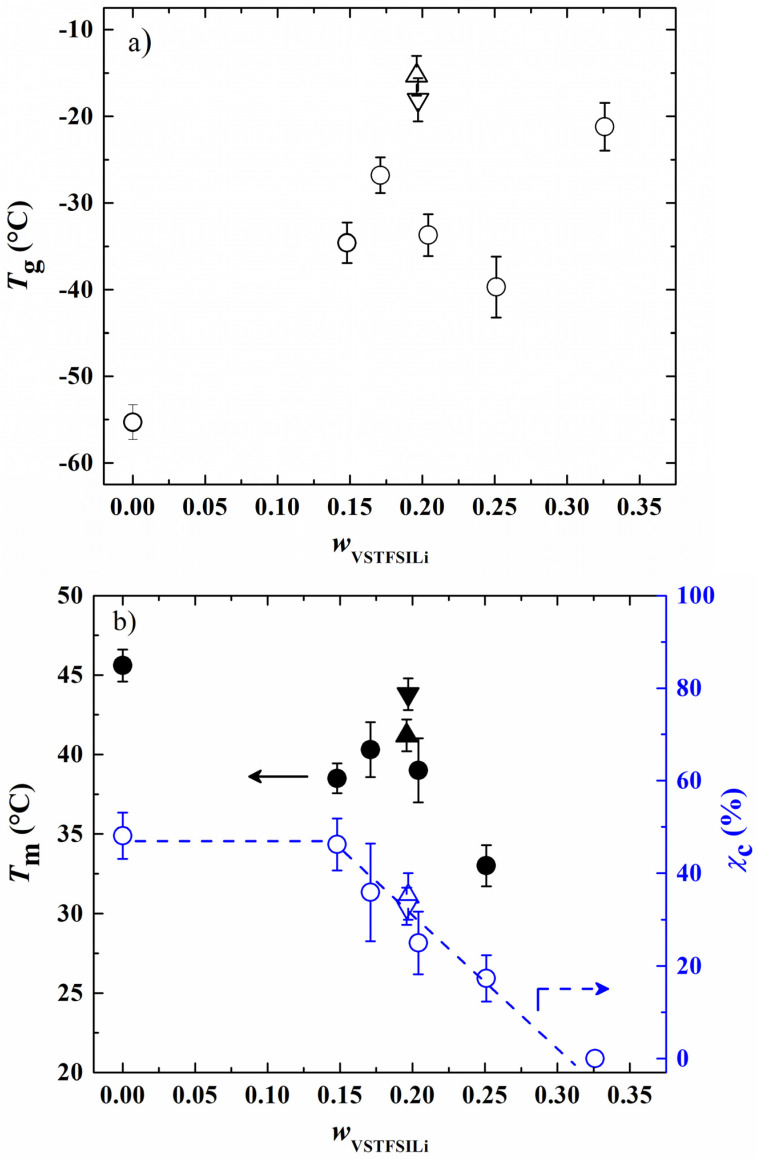
(**a**) Glass transition temperature (*T*_g_), and (**b**) melting temperatI (*T*_m_) and degree of crystallinity (χ_c_) as a function of *w*_VSTFSILi_. The circle symbols correspond to the HySI_*w*_VSTFSILi_ electrolytes while the up and down triangles correspond to HySI_20_TEOS and HySI_20_TEMS, respectively. In figure (**b**), filled and open symbols correspond to *T*_m_ and χ_c_, respectively. The dash lines are eye guides.

From each thermogram, *T*_g_ and *T*_m_ were determined as well as the degree of crystallinity (χ_c_, Equation (2)). In Figure 4a,b, *T*_g_, *T*_m_ and χ_c_ were plotted as a function of weight fraction of vinyl-STFSILi (*w*_VSTFSILi_). Globally, the *T*_g_ of studied HySI has the tendency to increase with the lithium content from −55.3 °C up to −21.2 °C in HySI_0 and HySI_33, respectively. Such a trend is similar to that encounter in PEO-based dual ion-conducting polymer electrolytes where increasing the salt content typically leads to an increase in *T*_g_ [31,32,33]. Indeed, the Li cations are typically coordinated by several EO sites, forming several ionic bonds that reduce the PEO chain dynamic and thus increase *T*_g_. For 14.8 < *w*_VSTFSILi_ < 25.1%, *T*_g_ fluctuates around an average value of −33.7 ± 5.3 °C. Two competitive effects may explain this behavior; the *T*_g_ dependance with the lithium content and the effect of the inorganic SiO_1.5_ crosslinking sites on the mobility of the EO segments, the microstructure of the materials. As mentioned in the synthesis paragraph, by fixing the molar ratio *r*_2_ = 1, the content of silica and lithium in HySI electrolytes is intimately linked together. Thus, it is difficult to distinguish the influence of these two effects. Therefore, the HySI_20_TEOS and HySI_20_TEMS that possess double the silica content were prepared, and their *T*_g_ value was compared to that of HySI_20 (Figure 4a). The *T*_g_ of the HySI_20_TEOS (−18.1 °C) and HySI_20_TEMS (−15.3 °C) samples are 15 to 18 °C higher than that of HySI_20 (−33.7 °C). For a given lithium content, an increase in the silica concentration, and thus the crosslinking sites, increases the PEO chain’s rigidity and hence its *T*_g_. This result seems to demonstrate that the SiO_1.5_ content pilots the dynamic of the PEO chains and affects the *T*_g_ values of HySI electrolytes. This influence was less pronounced in samples having silica fractions lower than 4 wt% (Table 2, entries 2–4 and 7) since the *T*_g_ of these samples hardly varies around an average value of −33.7 °C. 

In Figure 4b, *T*_m_ decreases with *w*_VSTFSILi_ between 45.6 (*w*_VSTFSILi_ = 0 wt%) and 33 °C (*w*_VSTFSILi_ = 25 wt%) while there is no *T*_m_ for the HySI_33 (*w*_VSTFSILi_ = 32.6 wt%) electrolyte. Such dependencies, already reported in the literature by several authors, [31,32,33] have been attributed to the coordination of the cation by ether oxygens of PEO. In addition, a smooth maximum at *w*_VSTFSILi_ of 17% is observed for the salty HySI electrolytes. In the article, the word salt designates the crosslinked VSTFSILi on the polymer matrix to create the single-ion behavior. Herein, since PEO chains are covalently bonded to the 3D silica networks, this can affect the crystallization of PEO due to the restriction imposed by the silica network. Indeed, the melting temperatures of HySI_20_TEOS and HySI_20_TEMS are higher than the one of HySI_20. Moreover, similarly to PEO-based electrolytes, χ_c_ decreases linearly with *w*_VSTFSILi_ from 46.2% (HySI_15) down to zero (HySI_33). Because of their high *T*_g_ and *T*_m_ values (less interesting for conductive property), HySI_20_TEOS and HySI_20_TEMS samples were not further studied.

**Figure 5:**
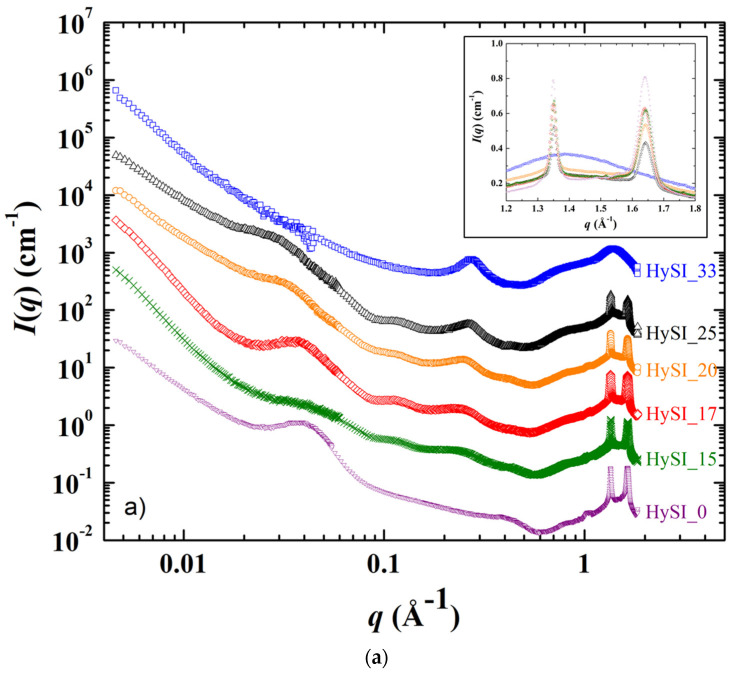

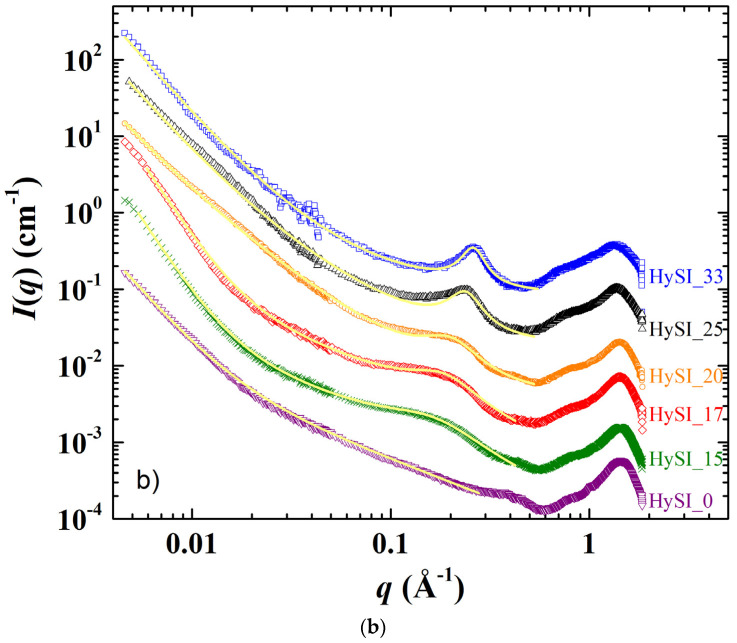
SAXS/WAXS scattering curves recorded at (**a**) 25 °C and (**b**) 60 °C for the HySI_*w*_VSTFSILi_ electrolytes with *w*_VSTFSILi_ of (▽) 0, (✕) 15, (◇) 17, (◯) 20, (△) 25, and (☐) 33. The curves are shifted in intensity for clarity. The inset in figure a) is a magnification in the high *q* range. The yellow continuous lines in figure (**b**) are the fit curves from the ad hoc model (see main text).

The SAXS/WAXS scattering patterns displaying the scattering intensity *I*(*q*) as a function of the scattering vector *q* of the electrolytes and of the salt-free sample at 25 °C and 60 °C are shown in Figure 5a,b. In Figure 5a, the scattering pattern from most of the samples can be divided into four domains over the whole *q* range: (i) at the lowest *q*, a scattering decay like *q*^−α^ with α in between −3 and 4; (ii) a broad correlation peak around 0.035 Å^−1^, followed by other well-defined peaks at larger *q* in the 0.05–0.2 Å^−1^ range for all samples except HySI_33; (iii) a correlation peak at ~0.26 Å^−1^, except for the salt-free sample (HySI_0); and (iv) two crystalline peaks at very large q* at 1.35 and 1.64 Å^−1^, except for HySI_33 sample where only a large amorphous diffuse ring arises.

As the HySI_33 sample is the only one that does not crystallize at 25 °C (see Figure 3b), the absence of the diffraction peaks (at q* = 1.35 and 1.64 Å^−1^) and correlation peaks at intermediate *q* in the 25 °C scattering curve (Figure 5a) demonstrates that such structural features come from the PEO crystalline structure. Indeed, they completely disappear when the electrolytes are heated at 60 °C, i.e., above their melting temperature (Figure 5b). The crystalline peaks correspond to distances in the crystallized PEO of 4.65 Å and 3.8 Å [34]. The large correlation peak at ~0.035 Å^−1^ and the other correlation peaks at intermediate *q* originate from the lamellar stacking of the crystallized PEO; the main peak being the first order peak and the other ones its harmonics. The exact positions of such peaks are difficult to precisely determine as they are superimposed with the overall scattering decay at law *q*, which varies from one sample to another, and with the broadening of correlation peak at 0.26 Å^−1^. Nevertheless, the distance between lamella is estimated to be about ~180 Å (2π/0.035). In addition, the intensity of the crystalline peaks decreases with increasing salt content. They are similar for HySI_15 and HySI_17 and progressively broaden for HySI_20 and HySI_25 (see inset of Figure 5a). The crystalline peak intensity is highest for the sample without salt which means that the presence of salt reduces the PEO crystallinity. This result is in accordance with the decrease of the degree of crystallinity χ_c_ with *w*_VSTFSILi_ calculated from DSC (see Figure 4b).

The data at 60 °C (Figure 5b) enable the identification of the structural features that are not associated with the PEO crystalline parts, namely a scattering decay at the lowest *q* following a power law ranging between *q*^−3^ and *q*^−4^ (for *q* < ~0.04 Å^−1^), another scattering decay with a much smaller lower power law (*q*^−1.7^ − *q*^−1^) at intermediate *q*, and the correlation peak at ~0.26 Å^−1^. The scattering decay at the lowest *q* comes from some fluctuations/heterogeneities of the electron-scattering length density within the samples. It is difficult to assess their exact origin as the samples contain three types of scatterers: PEO chains, grafted TFSI anions and silica crosslinkers. Given that both TFSI anions and silica species are located at the PEO chain ends and are the only species that contain atoms having a mass significantly larger than those of oxygen (sulfur for TFSI and silicone for silica), it is reasonable to assume that the scattering obtained at low *q* comes from the existence of domains either rich in silica and/or TFSI or rich in PEO. In other words, such scattering originates from the structure of the crosslinkers network. Indeed, it is also present for the salt-free sample that does not contain TFSI anions, demonstrating that the silica crosslinked nodes are not distributed evenly within the materials at a large scale. The value of the α exponent of the *q*^−α^ decay is linked to the morphology of the crosslinkers network. For the salt-free sample (HySI_0) and for the electrolytes with the higher salt concentration (HySI_20, HySI_25, and HySI_33), α equals 3 which reveals the presence of strong inhomogeneities within the sample. When α = 4, as for HySI_15 and HySI_17, we recover a typical Porod scattering that comes from the surface scattering of 3D objects. In these electrolytes, the PEO domains likely reach a large size, i.e., the sample displays locally a spatial phase separation. This is consistent with the fact that the PEO crystallite domains are the largest at 25 °C for such samples, as evidenced by the low width and high intensity of the crystalline peaks at large *q* (see inset Figure 5a). Remarkably, the low *q* scattering does not vary upon a 25–60–25 °C temperature cycle as shown in Figure S6, demonstrating that the electrolyte structure at a mesoscopic scale does not evolve upon PEO crystallization or melting. Indeed, over the temperature cycle, the PEO chains recrystallize in a similar fashion, as the 25 °C SAXS spectra of the electrolytes after heating at 60 °C superimposes, also at high *q*, with those recorded at 25 °C before heating. This indirectly demonstrates the high mechanical strength of the solid network, as a fragile loose network would have probably reorganized itself upon the formation/disappearance of PEO spherulites.

The other important structural feature is the correlation peak in the 0.1–0.3 Å^−1^ range for all electrolytes except the salt-free one (HySI_0), showing that the grafted TFSI induces the structuration at a local scale with a given characteristic size. Such a peak is intense at a well-defined position q* at 0.26 Å^−1^ for HySI_33, then progressively broadens and shifts towards lower *q* when decreasing salt content. In order to receive more quantitative insight into the structural parameters of the different samples, the SAXS scattering data at 60 °C were fitted with an ad hoc model over the entire *q* range, from 0.006 Å^−1^ to 0.5 Å^−1^, composed of the three contributions described above. The intensity was fitted by the sum of three terms: two power laws to describe the intermediate and low *q* regions (*I*~*q*^−α^ or *q*^−β^) with their respective exponents α and *β* and the correlation peak at large *q* by a Lorentzian distribution enabling the determination of its position $q_{0}$ and width λ. In addition, for the salt-free sample, only the two power laws were considered. The equation of the ad hoc model is thus:

(8) $$I\left({\mathrm{cm}}^{-1}\right)=\frac{A}{\pi \lambda \left[1+{\left(\frac{q-q_{0}}{\lambda }\right)}^{2}\right]}+\frac{B}{q^{\beta }}+\frac{C}{q^{\alpha }}$$

where *I* is the normalized scattered intensity; A, B and C are pre-factors of the Lorentzian term; *β* and *α* are exponents of power laws at low and intermediate *q* range, respectively; and $q_{0}$ and λ represent the peak position and peak broadening parameters for Lorentzian distribution. 

**Figure 6:**
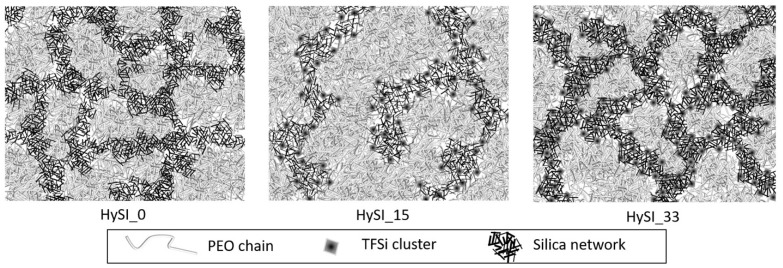
Schematic representation of hybrid-electrolyte network at 60 °C for HySI_0, HySI_15 and HySI_33. The gray background represents the PEO, the darker paths represent the silicic bridges and the black points the salt vinyl-TFSI crosslinked with the silica on the PEO chains.

The results of the fits are also added in Table S1. For the correlation peak, it gives a well-defined structure (*λ* = 0.038 Å^−1^) with a characteristic size *d*_0_ (=2π/*q*_0_) of 24.15 Å in direct space for the highest concentrated electrolyte (HySI_33), then *d*_0_ increases continuously with the decrease of the salt content up to 47.6 Å for HySI_15, associated with an increase in the broadening up to *λ* = 0.105 Å^−1^. In the crosslinked electrolyte, the emergence of this characteristic size originates from the differences of electron scattering length density in the three-component system (amorphous PEO with only light atoms, TFSI and SiO_1.5_ with Si and S heavier atoms, S being those that scatter most). The samples for which such a peak is visible contain mainly PEO and TFSI (15–33 wt.%), the silica content being rather low (below 4.2 wt.%). Given that the correlation peak only arises with TFSI, it is likely that this peak comes mainly from correlations between TFSI molecules, either individually and/or most probably in cluster domains. This is consistent with the electrolytes comprising the larger TFSI content that induce shorter distances in real space. As the VSTFSILi/silica network would have a homogenous 3D organization, they would be separated by a typical mean distance *d_mean_* that would scale with the volume density of molecules *ρ*_TFSI_ such as *ρ*_TFSI_ ~ 1/*d_mean_*^3^ due to dilution. Therefore, Figure S7 represents 1/*d*_0_^3^, with *d*_0_ obtained from SAXS fitting, as a function of *w*_VSTFSILi_. The behavior is linear suggesting a rather homogenous distribution of VSTFSILi/Silica clusters within the TFSI-rich domains. At last, the silica content in the hybrid materials seems to have little influence on the TFSI network structure. Indeed, the whole structure of the HySI_20_TEOS and HySI_20_TEMS are very close to those of the HySI_20, both at 25 °C and 60 °C (see Figure S8). Finally, to illustrate these findings, a schematic network of hybrid electrolytes is proposed in Figure 6. For small values of *w*_VSTFSILi_, such as HySI_15, larger PEO domains are observed with a distance inter-islet around 50 Å, and finally for the highest concentrated electrolytes, such as HySI_33, smaller PEO domains are observed with a distance inter-isled around 25 Å. The islets of silicic bridges crosslinked are more or less interconnected during the crosslinking process, and the PEO phase does not change the microstructure. In fact, the nanostructure of the HySI does not change before and after heating at 60 °C. The degree of local organization decreases in proportion to the decrease in silica and in salt as a consequence. The presence of a rich-PEO phase and of the rich-TFSI phase containing the silica (TFSI/SiO_1.5_) at 60 °C in the HySI shows a mesostructured network, so the impacts on the ionic transport properties are discussed.

**Figure 7:**
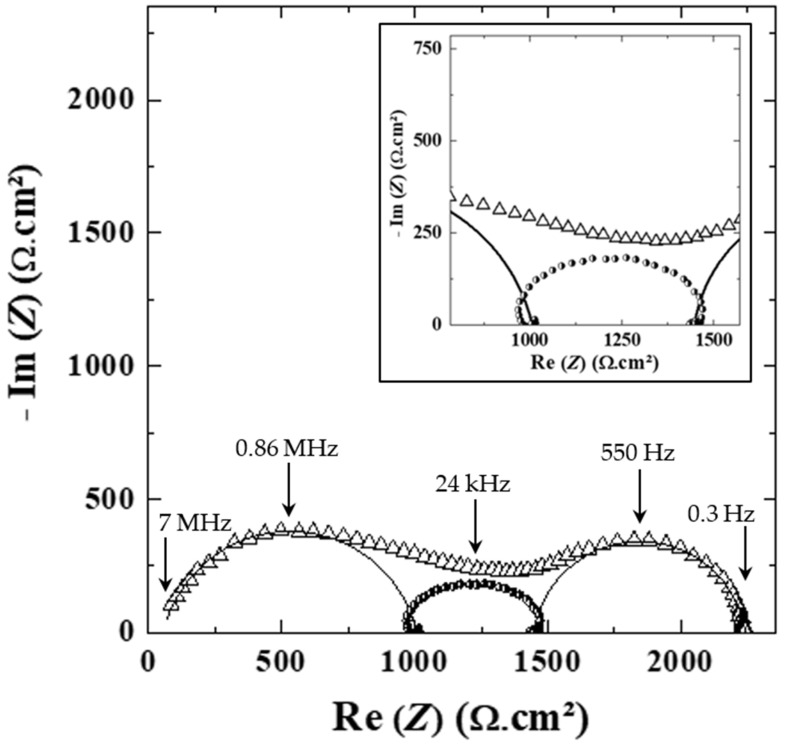
Impedance spectrum of the (△) HySI_25 electrolyte at 60 °C in Li symmetric cell. The fit of the contribution arising from the rich PEO phase (0.86 MHz) and the Li/electrolyte interface (550 Hz) are shown as a straight and dotted line, respectively. The contribution at MF (24 kHz, black circle) is obtained by the subtraction method (see text, TFSI/SiO_1.5_ phase contribution). The inset is a magnification in the MF range.

To analyze the ionic transport properties and the Li/electrolyte interface, electrochemical impedance spectroscopy (EIS) was performed on a series of Li symmetric cells comprising the HySI electrolytes. A typical EIS spectrum of the HySI_25 electrolyte recorded at 60 °C is presented in Figure 7. The experimental spectrum can be decomposed into two main frequency (*f*) domains with *f* > 4 kHz; a loop with a large frequency distribution from high to medium *f* is ascribed to the overall bulk contribution, followed by a clean arc of a circle at lower *f* corresponding to the Li/electrolyte interface (*f_c_* = 550 Hz) [35]. Those features are observed for all the EIS spectra of the electrolytes within the explored temperature range, i.e., from 30 °C to 100 °C. For completeness, Figure S9 displays the impedance spectra at 60 °C of the other HySI electrolytes with *w*_VSTFSILi_ of 15, 17, 20 and 33. The first loop starts with a clean arc of a circle (*f_c_* around 1 MHz) with a “tail” up to a few kHz with no clear contribution such as R//CPE; it cannot embed a sole process and can be at least decomposed into a high frequency (*f* > 100 kHz) and a medium frequency (100 < *f* < 4 kHz) contribution. Therefore, to obtain insight into the underlying contributions without arbitrary parameters, a methodology based on spectra differences [24] was employed to separate the different *f* domains at high (HF), medium (MF) and low (LF) frequency.

Firstly, the HF arc of circles is fitted with an electric equivalent circuit composed of resistance (*R*) in parallel to a Constant Phase Element (*CPE*): *R*//*CPE*. The result of the fit is shown in Figure S10a and Figure 7. This fitted HF contribution (*f_c_* = 0.86 MHz) is subtracted from the experimental data leading to a subtracted spectrum, shown in Figure S10b, comprising the remaining MF and LF contributions shifted by a constant value corresponding to the HF resistance. Then, the LF contribution is fitted by another R//CPE circuit (*f_c_* = 550 Hz), as shown in Figure S10b and Figure 7, and subtracted to the initial subtracted spectrum, leading to a remaining loop slightly depressed and deformed at HF and LF, which corresponds to the MF contribution as added in Figure S10c and Figure 7. It is worth noticing that this loop cannot be fitted properly by a simple R//CPE (see the inset of Figure 7, a zoom on this contribution at MF). Moreover, from this methodology, the characteristic frequencies at 60 °C taken as the apex frequencies of the HF, MF and LF domains are plotted as a function of *w*_VSTFSILi_ in Figure S11. For all the electrolytes, the three contributions are present. Therefore, this methodology based on successive subtraction steps permits a robust method to deconvolute different contributions with a large frequency distribution located within a close frequency range. 

To identify the main contributions related to the HF and MF processes, the dielectric constant (*ε_HF_*) of the HF contribution was calculated based on the results of the fits, listed in Table S2, using both resistance and constant phase element parameters (see Equation (5)). *ε_HF_* is on average 7.5 ± 1.0 with a slightly lower value for the highest concentrated electrolytes. In addition, the average value of *ε_HF_* is almost independent of the temperature. In the literature, the dielectric constant of PEO electrolytes ranges from 4 to 7.5 [36,37,38,39]. 

Moreover, based on the SAXS analysis (see Figure 5a,b), the HySI electrolytes are heterogeneous compounds at the meso/microscale with the presence of PEO- and TFSI/SiO_1.5_-rich domains, the latter comprising the salt and the silica crosslinked nodes. We thus attribute the HF (0.86 MHz) contribution to the major PEO-rich phase, and the following complex contribution at MF (24 kHz) to the presence of the minor TFSI/SiO_1.5_ domains. The co-existence of those two phases leads to the convoluted contributions observed in the impedance spectra for *f* > 4 kHz as the Li^+^ cation has to transfer through the PEO and the TFSI/SiO_1.5_ rich domains.

As during ionic transport, the ions move through the PEO- and TFSI/SiO_1.5_- rich domains. According to the microstructure, we assume that they behave as in nanostructured ceramics with the grain (PEO-rich) and alternate grain boundaries (TFSI/SiO_1.5_-rich) [40], the two contributions being then considered in series. The effective conductivity (*σ*_t_) of the HySI can be calculated based on Equation (3), considering that the effective resistance of the membrane is the sum of the HF and MF resistances. In addition, one can calculate the apparent ionic conductivity due to both contributions normalizing the resistances by the geometrical factor of the membrane, such as:

(9) $$\frac{1}{\sigma_{t}}=\frac{1}{\sigma_{\mathrm{PEO}}}+\frac{1}{\sigma_{\mathrm{TFSI}/{\mathrm{SiO}}_{1.5}}}$$

**Figure 8:**
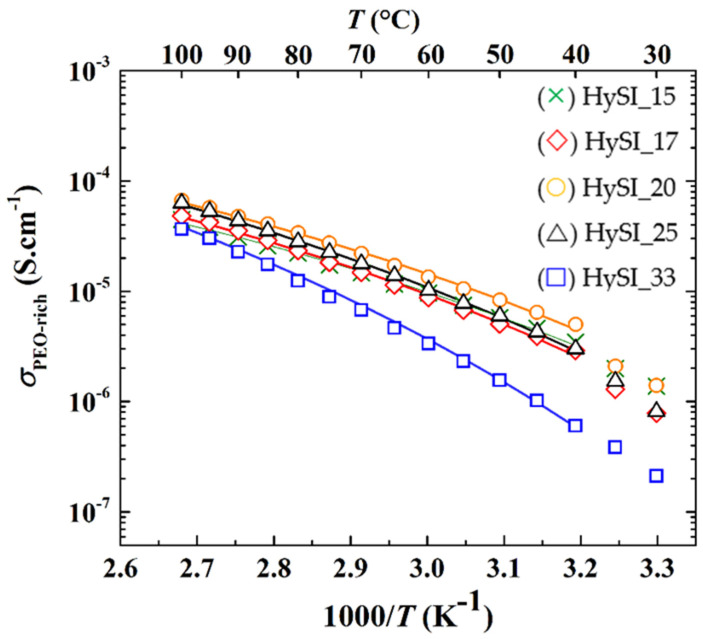
Ionic conductivity of the PEO-rich as a function of the inverse of the temperature for HySI_*w*_VSTFSILi_ electrolytes. The lines correspond to the VTF fits.

The apparent ionic conductivity of the PEO (*σ*_PEO_) is then plotted as a function of the inverse of the temperature in Figure 8. The conductivity of the PEO phase of the HySI electrolytes follows a typical VTF behavior for PEO-based electrolytes [41], with a break at about 40 °C corresponding to *T*_m_ (see Figure 4b), except for HySI_33 as this electrolyte remains amorphous in the explored temperature range. In addition, the conductivity of the PEO phase of the HySI_33 electrolyte remains lower than all the other compositions. Except for HySI_33, *σ*_PEO_ ranges from about 4 × 10^−6^ to 5 × 10^−5^ S·cm^−1^ at 40 and 100 °C, respectively. These values are similar to those encountered in single-ion conductor block copolymer electrolytes based on PEO but synthesized via a more complex, multi-step and time-consuming route [42]. The ionic conductivity of the PEO-rich phase in the amorphous state was then fitted using the VTF Equation (4). The ideal glass transition temperature (*T*_0_) is taken as being *T*_g_—50 while the two other parameters of the VTF equation, *σ*_0_ and *E*_a_, were left free. The results of the best fit (*χ*^2^ > 0.99) are added to Figure 8, showing the good agreement between experimental and fitted data. For completeness, the fitted parameters, *σ*_0_ and *E*_a_, are plotted as a function of *w*_VSTFSILi_ in Figure S12. For *w*_VSTFSILi_ < 0.25, the pseudo-activation energy is close to that of PEO/LiTFSI electrolytes [43] of about 7.8 kJ·mol^−1^ while the pre-exponential factor is in the same range as those found in single-ion conductor block copolymer electrolytes based on PEO [42]. These results confirm that the ionic transport mechanism in HF contribution of the crosslinked hybrid electrolyte is similar to that encountered in PEO-based electrolytes. At a higher salt content, with *w*_VSTFSILi_ of 0.25 and 0.33, the VTF parameters deviate from that of PEO-based electrolytes with a steady increase in *σ*_0_ while *E*_a_ tends to reach a plateau value at about 11.2 kJ·mol^−1^. Such increases in *E*_a_ with the number of crosslinked nodes are also reported in other materials in the literature [44,45]. Indeed, at higher concentrations, while there is a higher fraction of free ions (*σ*_0_ in Figure S12) to move within the electrolyte, the ionic transport via the movements of the polymer chain is more difficult. This can be related to the presence of large TFSI domains comprising the silica crosslinked nodes (see Figure 6).

**Figure 9:**
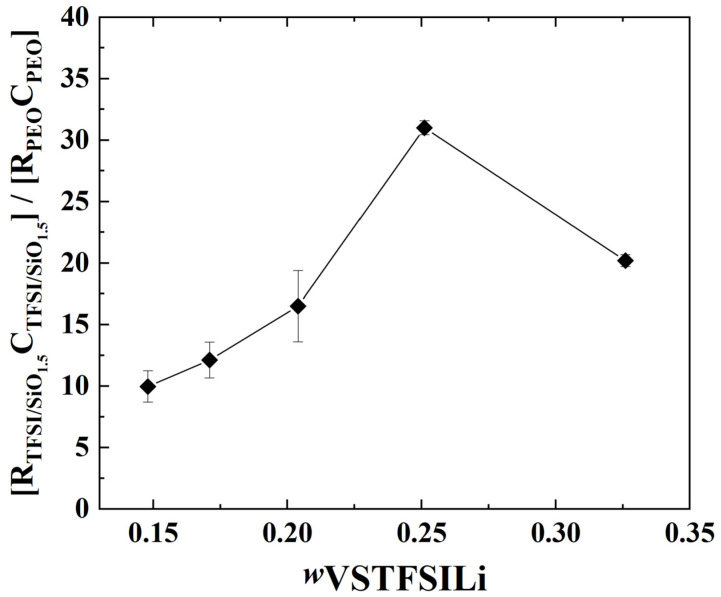
Ratio of the resistance *R*, multiplied by the capacitor *C* (fits results from EIS), of the phases TFSI/SiO_1_.-rich on PEO-rich at 60 °C as a function of *w*_VSTFSILi_. The lines are guidelines for the eyes.

Unfortunately, the precise geometrical factor of each phase (TFSI and PEO rich) cannot be determined accurately, especially for the minor TFSI/SiO_1.5_-rich domain; however, at a given temperature, the characteristic frequency $f_{c}$ at the apex of each material contribution is proportional to the ratio σ_ι_ over dielectric constant $\varepsilon_{r}$ according to: $f_{c}=\frac{\sigma_{i}}{2\pi \varepsilon_{r}\varepsilon_{0}}$. If we assume that the dielectric properties of the two phases are similar [46], the ratio of the $f_{c}$ for the PEO-rich and TFSI/SiO_1.5_-rich domains is proportional to the ratio of the true conductivity of each domain, PEO-rich and TFSI/SiO_1.5_-rich *f*_c_(HF)/*f*_c_(MF) = *σ*(HF)/*σ*(MF). Herein, a factor between 10 and 30 is obtained at 60 °C from the EIS fits (see Figure 9); that ratio decreases linearly with the TFSI/SiO_1.5_ wt%, which suggests less blocking behavior of the MF contribution at a higher TFSI/ SiO_1.5_ concentration. This increase may be due to the specific microstructure change as a function of the SiO_1.5_ wt.% and VSTFSILi wt.%, as illustrated in Figure 6. 

**Figure 10:**
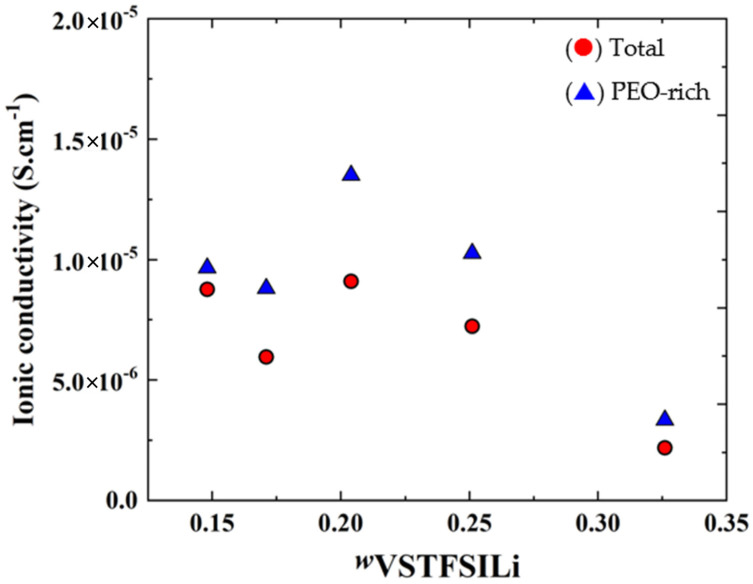
Isothermal variations at 60 °C of the (●) total conductivity *σ*_t_ containing PEO-rich phase with TFSI-rich domains and (▲) PEO-rich phase *σ*_PEO_, as a function of *w*_VSTFSILi_.

Figure 10 represents the isothermal conductivities at 60 °C of the HySI (total, effective), PEO-rich domains and as a function of *w*_VSTFSILi_. As seen in Figure 8, *σ*_PEO_ has a smooth dependence on the salt content with a maximum *w*_VSTFSILi_ of 0.204. This material is among the best dry single-ion polymer electrolytes with a good compromise between low cost, easy scale-up synthesis, and good mechanical properties [35,41,47]. *σ*_Total_ also has a smooth dependency with *w*_VSTFSILi_ due to a microstructural effect, such as an increase of the density and fractal dimension from 1D to 2D of the TFSI/ SiO_1.5_ network. 

Finally, the cationic transference number (*t*^+^) was determined at 80 °C for each HySI electrolyte using electrochemical methodologies applied to Li symmetric cells based on a chronoamperometric experiment (see Figure S1). For *t*^+^, the PEO and TFSI/SiO_1.5_ phases were considered as a whole that drives the effective conductivity of the materials, i.e., the electrolyte resistance to consider in Equation (7) is the sum of *R*_PEO_ and *R*_TFSI_. 

**Figure 11:**
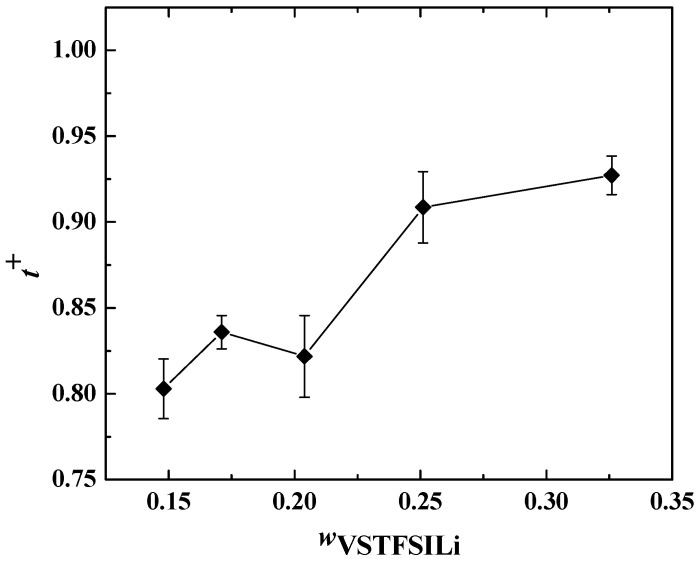
Cationic transference number at 80 °C as a function of *w*_VSTFSILi_. The lines are guidelines for the eyes.

In Figure 11, *t*^+^ is plotted as a function of *w*_VSTFSILi_ at 80 °C. *t^+^* remains constant at low salt concentrations for *w*_VSTFSILi_ < 0.25, with an average value of 0.83 ± 0.02, then becomes higher than 0.9 for higher salt content. Such values are consistent with the literature data on single-ion conducting polymer electrolytes [32,42,48,49]. Theoretically, *t*^+^ equals 1 as, by design, the TFSI anions are grafted onto the crosslinked nodes. However, experimentally, the cationic transference number of single-ion conducting polymer electrolytes is typically between 0.8 and 1 [30]. This effect is mainly ascribed to the stability of the passive layers at the Li/electrolyte interface playing a role in the interfacial resistance and the steady-state current used to calculate *t*^+^ (Equation (7)) [50]. 

## 4. Conclusions

Homogenous hybrid organic–inorganic solid single-ion electrolytes based on a PEO/silica network with attached TFSI were successfully synthesized by combining a Michael addition reaction, an epoxidation reaction and sol–gel polycondensation. SAXS investigations at 25 °C and at 60 °C demonstrated the existence of mesostructuration within hybrid electrolytes with domains rich in lithium salt around the silica nodes and domains poor in lithium salt far from these silica nodes. These silica nodes become a connected network with the increase of TFSI/silica content. The crosslinking is very homogeneous and forms nodes well-distributed in the material. The mesostructuration at 25 °C does not change after heating at 60 °C. A subtractive method was proposed to analyze the impedance spectra which indicates clearly, in agreement with the SAXS analysis, the contribution of two domains, with highly conductive domains rich in PEO and less conductive domains rich in TFSI/SiO_x_. The large distribution of frequencies leads to determining the apparent conductivity of each contribution, and the dielectric constant at HF is attributed to the PEO. The ionic transport properties are very good above melting while preserving a single ion Li^+^ conductivity paired with the standard dry SIPEs. In addition, with this new synthetic approach, it is easy to modulate the lithium concentration, the PEO and the silica content, and hence the mechanical and thermal properties of the resulting electrolyte materials. The favorable mechanical properties and thermal stability are useful for the practical application of hybrid electrolytes.

## Figures and Tables

**Table 1:** Experimental data used for the synthesis of HySI electrolytes.

Entry	Samples	EO/Li	m_APTES_, g (n, mmol)	m_VSTFSILi_, g (n, mmol)	m_PEO_, g (n, mmol)	r_1_^c^	r_2_^d^
1	HySI_0	-	0.0753 (0.340)	0 (0)	0.5300 (0.129)	/	2.64
2	HySI_15	30	0.0824 (0.372)	0.1100 (0.449)	0.5923 (0.144)	1.66	1.01
3	HySI_17	25	0.0966 (0.436)	0.1380 (0.563)	0.6192 (0.151)	1.55	1.01
4	HySI_20	20	0.1060 (0.479)	0.1630 (0.665)	0.5851 (0.142)	1.44	1.01
5 ^a^	HySI_20_TEOS	20	0.1060 (0.479)	0.1630 (0.665)	0.5851 (0.142)	1.44	1.01
6 ^b^	HySI_20_TEMS	20	0.1060 (0.479)	0.1630 (0.665)	0.5851 (0.142)	1.44	1.01
7	HySI_25	15	0.1207 (0.545)	0.2010 (0.820)	0.5411 (0.132)	1.33	1.01
8	HySI_33	10	0.1436 (0.649)	0.2607 (1.064)	0.4679 (0.132)	1.22	1.00

In steps 1 and 2, 2 g of ethanol were used for each synthesis. ^a^ In step 3, 86.44 mg of tetraethoxysilane was added to the reaction mixture. ^b^ Additionally, in step 3, 94.27 mg of triethoxymethylsilane was added to the reaction mixture ^c^ The molar ratio between reactive functions of APTES and vinyl-STFSILi compounds with $r_{1}=\frac{2n_{APTES}}{n_{VSTFSILi}}$. ^d^ The molar ratio between reactive functions of APTES, vinyl-STFSILi and diepoxy–PEO compounds with $r_{2}=\frac{2n_{APTES}}{\left[n_{VSTFSILi}+2n_{PEO}\right]}$.

**Table 2:** Composition of the HySI electrolytes.

Entry	Electrolyte (HySI_*w*_VSTFSILi_)	*w*_VSTFSILi_ (wt.%)	*w*_SiO1.5_ (wt.%)	*w*_PEO_ (wt.%)	*w*_VSTFSILi_ /*w*_SiO1.5_	EO/Li
1	HySI_0	0	3.11	93.4	0	-
2	HySI_15	14.8	2.61	79.7	5.7	30
3	HySI_17	17.1	2.82	76.9	6.1	25
4	HySI_20	20.4	3.11	73.1	6.55	20
5 ^a^	HySI_20_TEOS	19.7	6.11	70.9	3.2	20
6 ^b^	HySI_20_TEMS	19.6	6.22	70.2	3.15	20
7	HySI_25	25.1	3.53	67.5	7.1	15
8	HySI_33	32.6	4.22	58.5	7.7	10

^a^ This sample contains 3.11 wt% of SiO_1.5_ species and 3 wt% of SiO_2_ species. ^b^ Sample synthesized with additional triethoxymethylsilane (TEMS).

## Data Availability

Data are available upon request to the corresponding authors.

## Supplementary Materials

The following supporting information can be downloaded at: https://www.mdpi.com/article/10.3390/polym14235328/s1, Table S1. Fit result of the ad hoc model based on Equation (8) (see main text). Table S2. Fit parameters of the PEO contribution at high frequencies (high-*f* or HF) portion of the EIS spectra of the electrolytes recorded at 60 °C comprising the resistance *R*_high-_*_f_*, the pseudo-capacity *Q*_high-*f*_ of phase *n*_high-*f*_ as well as the calculated capacity *C* and the dielectric constant of the bulk *Ɛ*_high-_*_f_*. Figure S1. (a) Evolution of the current with time during a constant voltage (20 mV) step at 80 °C on a Li symmetric cell comprising the HySI_33 electrolyte at 80 °C. (b) EIS spectra recorded before and after the polarization step. Figure S2. ^1^H NMR spectra of reactional mixture of APTES functionalized with lithium vinyl-TFSI Figure S3. (a) ^19^F NMR spectrum and (b) ^7^Li NMR spectrum of APTES functionalized with lithium vinyl-TFSI. Figure S4. Frequency response at 75 °C and at a strain value fixed at 1% of the storage (G′, filled symbols) and loss (G″, open symbols) modulus of the (triangle) HySI_0 and (circle) HySI_20 materials. Figure S5. DSC thermograms of the HySI_0, HySI_20-TEMS and HySI_20-TEOS electrolytes. Figure S6. SAXS/WAXS scattering curves for the HySI electrolyte during the 25–60–25 °C temperature cycle. Figure S7. 1/d_0_^3^ as a function of *w*_VSTFSILi_ of the HySI electrolytes. The line is a linear fit. Figure S8. SAXS/WAXS scattering curves for the HySI_20, HySI_20_TEOS, and HySI_20_TEMS electrolytes at 25 and 60 °C. Figure S9. Impedance spectrum of the HySI electrolytes recorded at 60 °C with *w*_VSTFSILi_ of (a) 15, (b) 17, (c) 20 and (d) 33 with three main contributions at HF, MF and LF. Figure S10. Impedance spectrum of the HySI electrolyte *w*_VSTFSILi_ = 25 recorded at 60 °C. The blue lines represent the fit of a specific contribution with (a) fit of the Bulk at HF and (b) fit of the interface Li at LF. (c) All the contributions are represented (HF, LF in blue and the MF contribution TFSI/SiO_1.5_ in green), obtained using a subtractive method between the initial impedance spectra and the fits at HF and LF. Figure S11. Apex frequencies at 60 °C of the three contributions of the impedance spectra at (✕) HF, (△) MF and (◯) LF as a function of *w*_VSTFSILi_ of the HySI electrolytes. Figure S12. VTF parameters, pre-exponential factor (*σ*_0_, filled symbols) and pseudo-activation energy (*E*_a_, open symbols) as a function of *w*_VSTFSILi_ for the PEO rich phase.
