# Supplementary material for: New Crosslinked Single-Ion Silica-PEO Hybrid Electrolytes
Source: Polymers (Basel). 2022 Dec 6;14(23):5328. doi: 10.3390/polym14235328 (PMC9735500; doi:10.3390/polym14235328)
Supplement: Supplementary file 1 [file polymers-14-05328-s001.zip › polymers-1980764-supplementary.pdf]

Supporting Information

New Crosslinked Single-Ion Silica-PEO Hybrid Electrolytes

Sébastien Issa [1,†], Roselyne Jeanne-Brou [2,†], Sumit Mehan [3], Didier Devaux [2], Fabrice Cousin [3], Didier Gigmes [1], Renaud Bouchet [2,] and Trang N. T. Phan [1,*]*

1 Institut de Chimie Radicalaire-UMR 7273, CNRS, Aix Marseille University, 13397 Marseille, France;

2 LEPMI, Grenoble INP, CNRS, Univ. Savoie Mont Blanc, Univ. Grenoble Alpes, 38000 Grenoble, France

3 Laboratoire Léon Brillouin, Université Paris-Saclay, CEA-CNRS UMR 12, 91191 Gif-sur-Yvette, France

* Correspondence: renaud.bouchet@grenoble-inp.fr (R.B.); trang.phan@univ-amu.fr (T.N.T.P.)

† These authors contributed equally to this work.

Electrolyte (HySI_$w_{VSTFSILi}$)	$w_{VSTFSILi}$ (wt. %)	A	q_0	λ	B	*β*	*C*	*α*
HySI_0	0				0.0369	1	9.39e-6	3
HySI_15	14.8	0.268	0.132	0.105	0.0270	1.11	1.65e-7	4
HySI_17	17.1	0.223	0.160	0.082	0.0230	1.11	1.99.e-7	4
HySI_20	20.4	0.249	0.190	0.073	0.0061	1.74	1.85e-5	3
HySI_25	25.1	0.182	0.235	0.049	0.0042	1.55	1.50e-5	3
HySI_33	32.6	0.215	0.259	0.038	0.0053	1.35	1.70e-5	3
HySI_20-TEOS	19.7	0.311	0.172	0.074	0.0079	1.40	1.67e-5	3
HySI_20-TEMS	19.6	0.209	0.190	0.085	0.0071	1.40	2.50e-5	3

Table S1. Fit result of the ad-hoc model based on equation (8) (see main text).

Electrolyte (HySI_$w_{VSTFSILi}$)	$w_{VSTFSILi}$ (wt. %)	$R_{high\text{-}f}$ (Ω)	$Q_{high\text{-}f}$ ($F.s^{(1-n)}$)	$n_{high\text{-}f}$	$C_{high\text{-}f}$ (F)	$\varepsilon_{high\text{-}f}$
HySI_15	14.8	780	$5.06\ 10^{-9}$	0.86	$8.36\ 10^{-11}$	8.4 ± 0.2
HySI_17	17.1	1180	$4.29\ 10^{-9}$	0.86	$6.19\ 10^{-11}$	7.2 ± 1.2
HySI_20	20.4	960	$4.61\ 10^{-9}$	0.85	$4.81\ 10^{-11}$	6.9 ± 0.4
HySI_25	25.1	1212	$4.09\ 10^{-9}$	0.86	$5.10\ 10^{-11}$	8.1 ± 0.5
HySI_33	32.6	2173	$4.28\ 10^{-9}$	0.87	$6.49\ 10^{-11}$	5.8 ± 0.8

Table S2. Fit parameters of the PEO contribution at high frequencies (high-*f* or HF) portion of the EIS spectra of the electrolytes recorded at 60 °C comprising the resistance $R_{high\text{-}f}$, the pseudo-capacity $Q_{high\text{-}f}$ of phase $n_{high\text{-}f}$ as well as the calculated capacity *C* and the dielectric constant of the bulk $\varepsilon_{high\text{-}f}$.

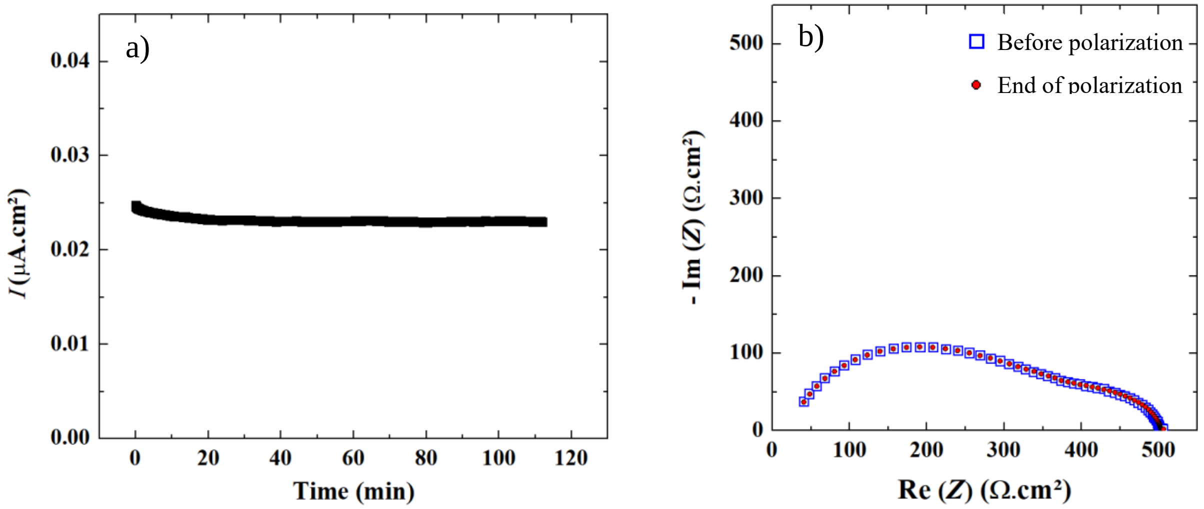

Figure S1. (a) Evolution of the current with time during a constant voltage (20 mV) step at 80 °C on a Li symmetric cell comprising the HySI_33 electrolyte at 80 °C. (b) EIS spectra recorded before and after the polarization step.

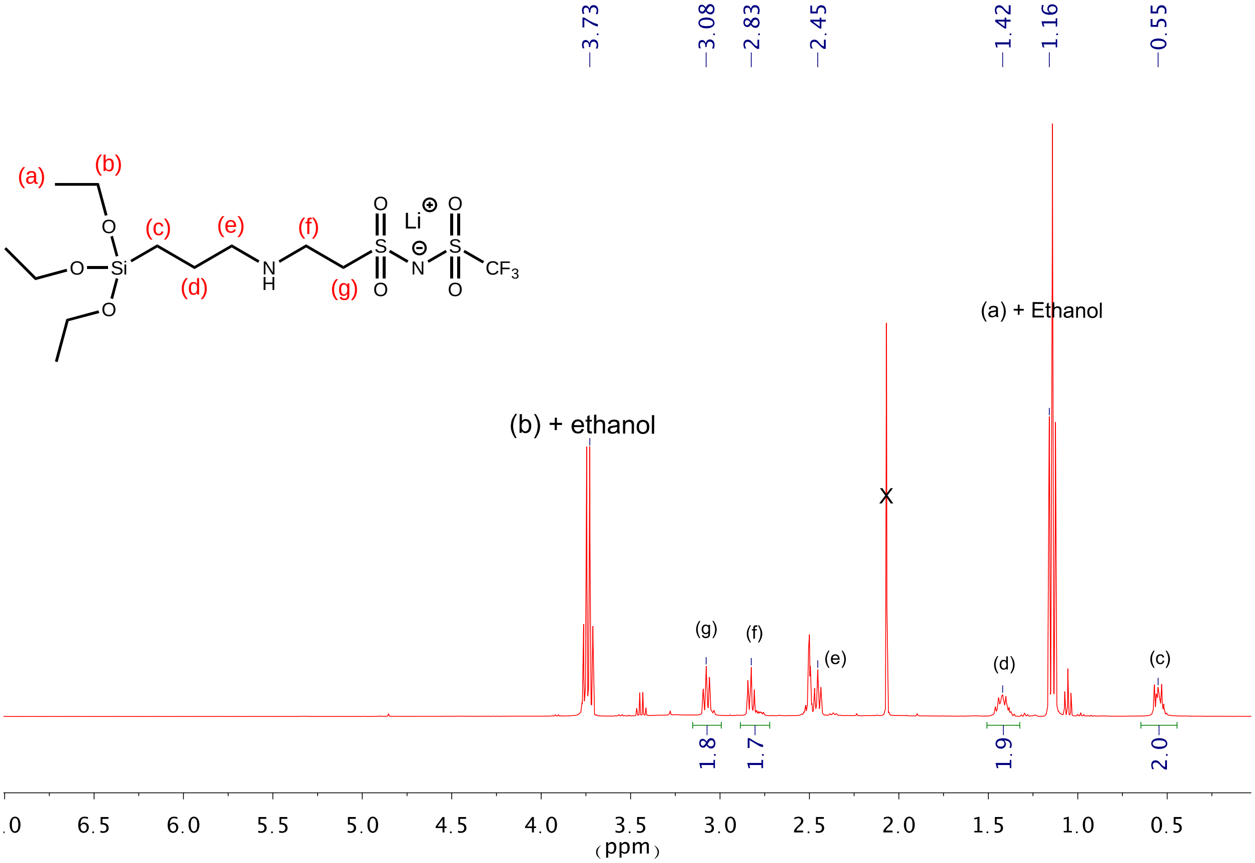

Figure S2. ^{1}H NMR spectra of reactional mixture of APTES functionalized with lithium vinyl-TFSI

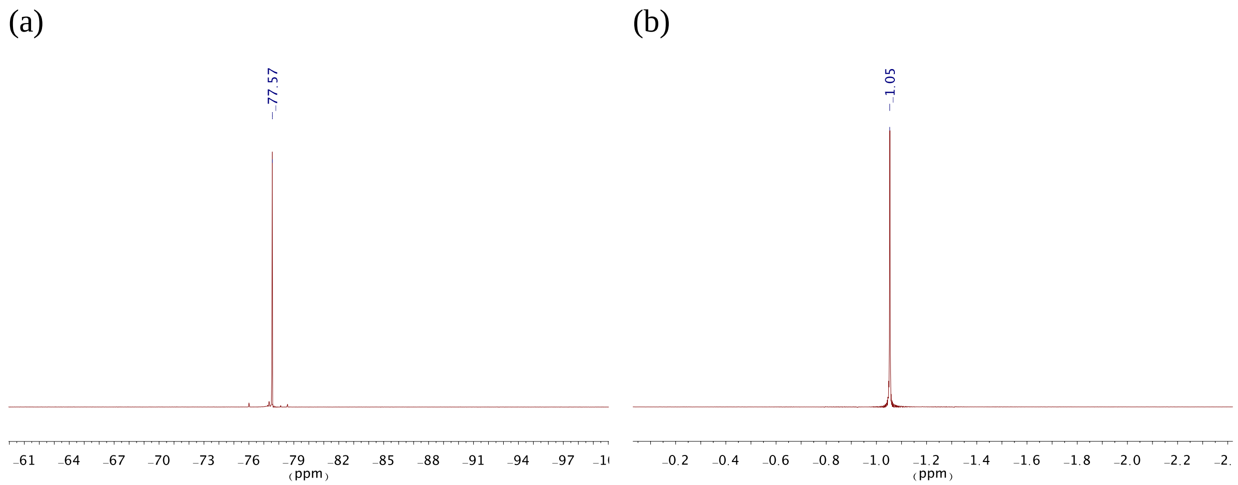

Figure S3. (a) ^{19}F NMR spectrum and (b) ^{7}Li NMR spectrum of APTES functionalized with lithium vinyl-TFSI.

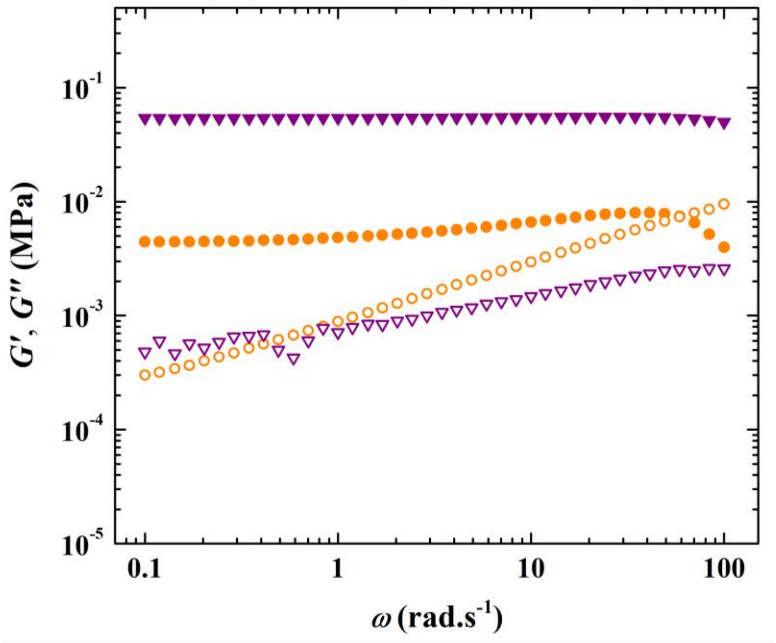

Figure S4. Frequency response at 75°C and at a strain value fixed at 1% of the storage (*G'*, filled symbols) and loss (*G''*, open symbols) modulus of the (triangle) HySI_0 and (circle) HySI_20 materials.

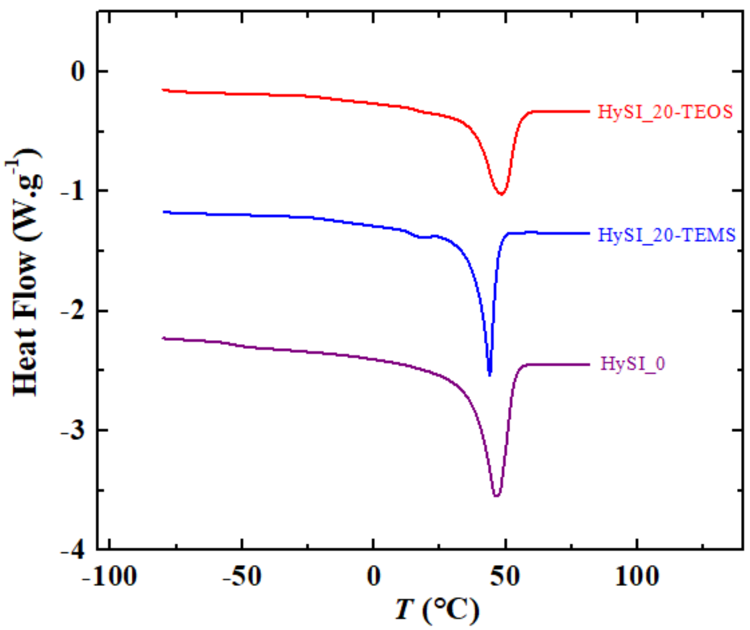

Figure S5. DSC thermograms of the HySI_0, HySI_20-TEMS and HySI_20-TEOS electrolytes.

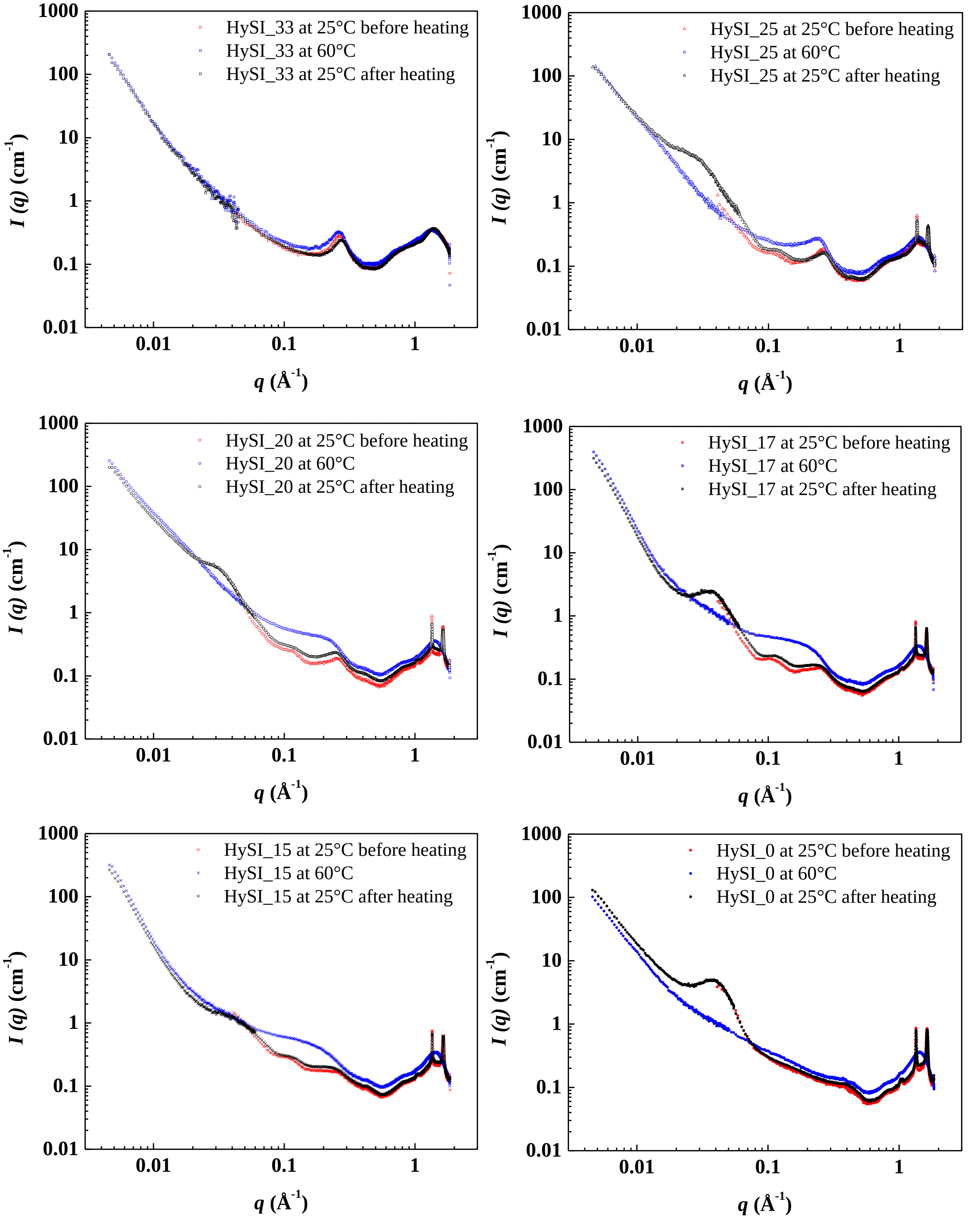

Figure S6. SAXS/WAXS scattering curves for the HySI electrolyte during the 25 - 60 - 25 °C temperature cycle.

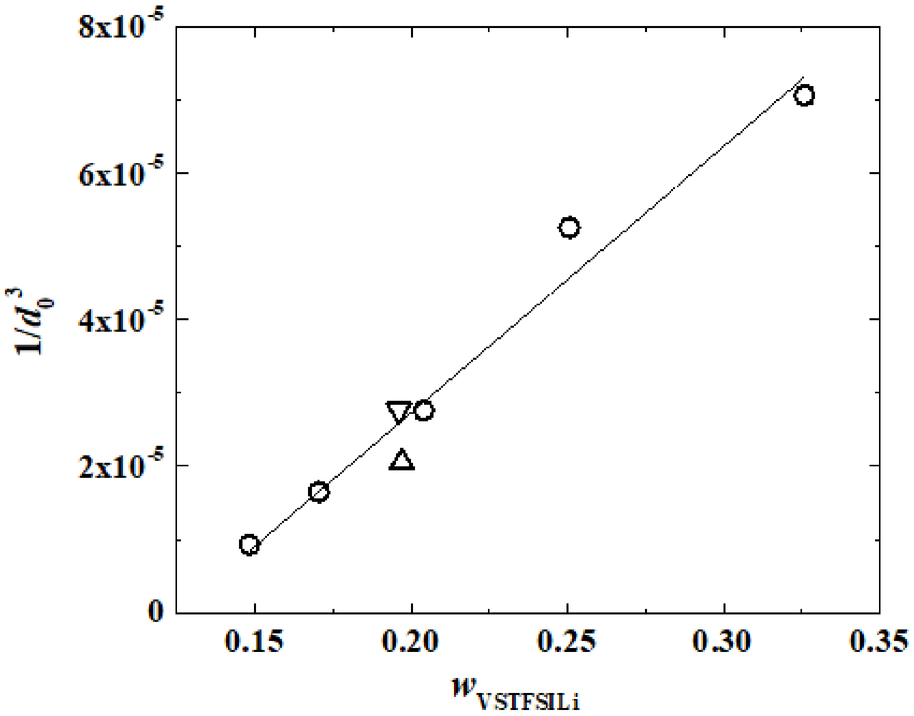

Figure S7. $1/d_0^3$ as a function of as a function of $w_{VSTFSILi}$ of the HySI electrolytes. The triangles correspond to the HySI_20_TEOS and HySI_20_TEMS. The line is the best linear fit.

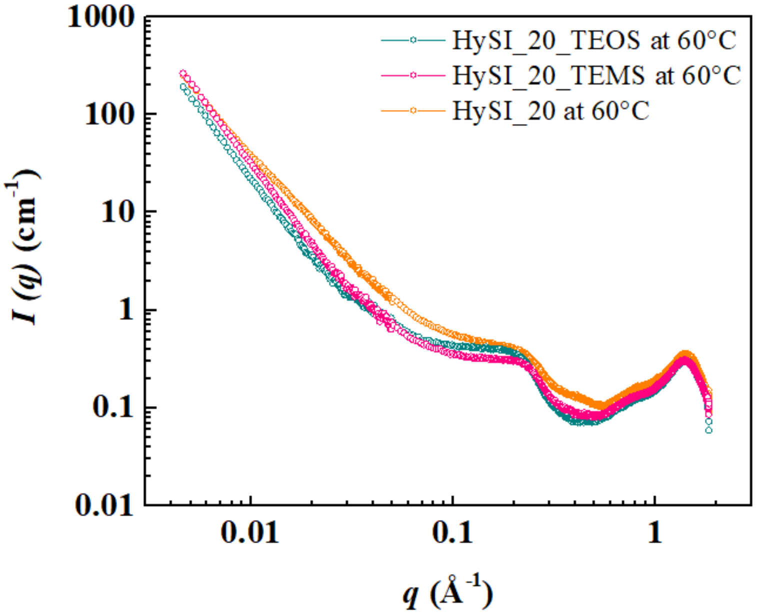

Figure S8. SAXS/WAXS scattering curves for the HySI_20, HySI_20_TEOS, and HySI_20_TEMS electrolytes at 25 and 60 °C.

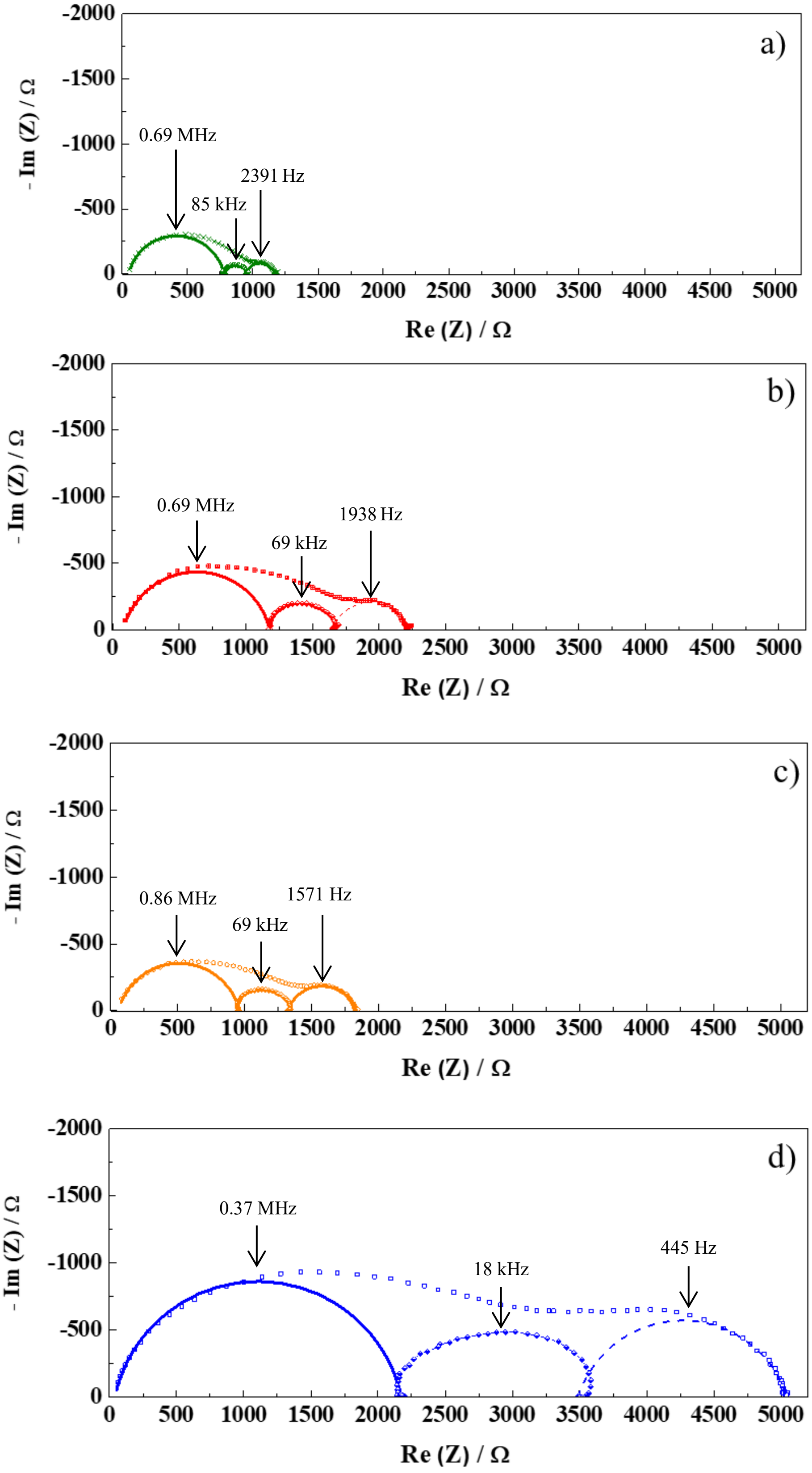

Figure S9. Impedance spectrum of the HySI electrolytes recorded at 60 °C with w_{VSTFSILi} of (a) 15, (b) 17, (c) 20, and (d) 33.

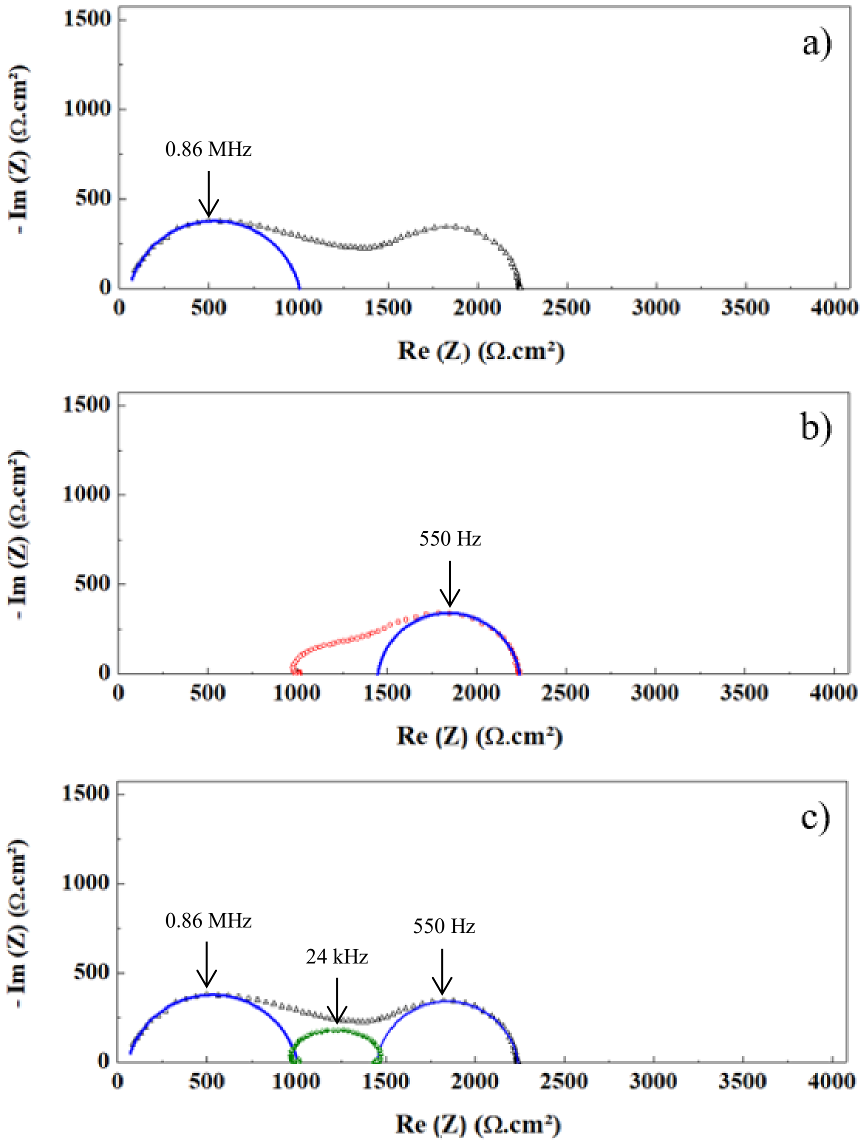

Figure S10. Impedance spectrum of the HySI electrolyte $w_{VSTFSILi}$ = 25 recorded at 60 °C. The blue lines represent the fit of a specific contribution with (a) fit of the Bulk at HF, (b) fit of the interface Li at LF, (c) all the contributions are represented (HF, LF in blue) and the MF contribution TFSI/$SiO_{1.5}$ in green obtained by a subtractive method between the initial impedance spectra and the fits at HF and LF.

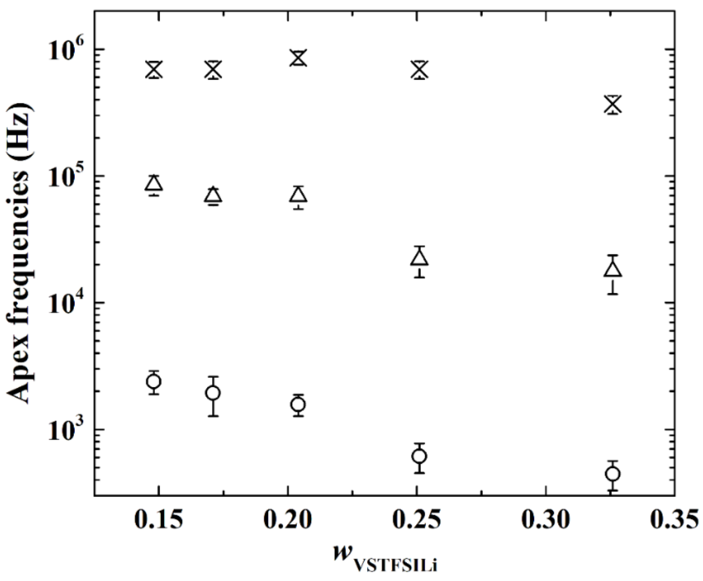

Figure S11. Apex frequencies at 60 °C of the three contributions of the impedance spectra at (×) HF, (△) MF, and (○) LF as a function of $w_{VSTFSILi}$ of the HySI electrolytes.

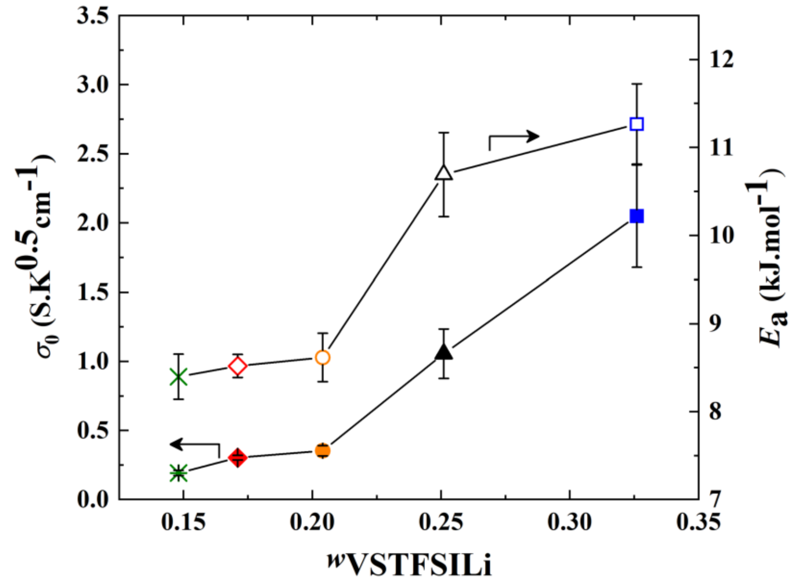

Figure S12. VTF parameters, pre-exponential factor (σ_0, filled symbols) and pseudo-activation energy (E_a, open symbols) as a function of $w_{VSTFSILi}$ for the PEO.
